# A roadmap for research in post-stroke fatigue: Consensus-based core recommendations from the third Stroke Recovery and Rehabilitation Roundtable

**DOI:** 10.1177/17474930231189135

**Published:** 2023-10-12

**Authors:** Coralie English, Dawn B Simpson, Sandra A Billinger, Leonid Churilov, Kirsten G Coupland, Avril Drummond, Annapoorna Kuppuswamy, Mansur A Kutlubaev, Anners Lerdal, Amreen Mahmood, G Lorimer Moseley, Quentin J Pittman, Ellyn A Riley, Brad A Sutherland, Connie HY Wong, Dale Corbett, Gillian Mead

**Affiliations:** 1 School of Health Sciences, College of Health, Medicine and Wellbeing, University of Newcastle, Callaghan, NSW, Australia; 2 Heart and Stroke Program, Hunter Medical Research Institute, Newcastle, NSW, Australia; 3 Department of Neurology, University of Kansas Medical Centre, University of Kansas Alzheimer’s Disease Research Centre, Kansas City, KS, USA; 4 Department of Medicine (RMH), University of Melbourne, Heidelberg, VIC, Australia; 5 School of Biomedical Sciences and Pharmacy, College of Health, Medicine and Wellbeing, University of Newcastle, Callaghan, NSW, Australia; 6 School of Health Sciences, University of Nottingham, Nottingham, UK; 7 Queen Square Institute of Neurology, University College London, London, UK; 8 Department of Neurology, Bashkir State Medical University, Ufa, Russia; 9 Department of Interdisciplinary Health Sciences, Institute of Health and Society, Faculty of Medicine, University of Oslo, Oslo, Norway; 10 Research Department, Lovisenberg Diaconal Hospital, Oslo, Norway; 11 Faculty of Health, Health and Education, Manchester Metropolitan University, Manchester, UK; 12 IIMPACT in Health, University of South Australia, Adelaide, SA, Australia; 13 Department of Physiology and Pharmacology, Hotchkiss Brain Institute, University of Calgary, Calgary, AB, Canada; 14 Department of Communication Sciences and Disorders, Syracuse University, Syracuse, NY, USA; 15 Tasmanian School of Medicine, College of Health and Medicine, University of Tasmania, Hobart, TAS, Australia; 16 Centre for Inflammatory Diseases, Department of Medicine, School of Clinical Sciences at Monash Health, Monash University, Clayton, VIC, Australia; 17 Department of Cellular and Molecular Medicine, University of Ottawa Roger Guindon Hall, Ottawa, ON, Canada; 18 Ageing and Health, Usher Institute, University of Edinburgh, Edinburgh, UK

**Keywords:** Stroke, rehabilitation, recovery, consensus, fatigue, measurement, mechanisms

## Abstract

**Rationale:**

Fatigue affects almost half of all people living with stroke. Stroke survivors rank understanding fatigue and how to reduce it as one of the highest research priorities.

**Methods:**

We convened an interdisciplinary, international group of clinical and pre-clinical researchers and lived experience experts. We identified four priority areas: (1) best measurement tools for research, (2) clinical identification of fatigue and potentially modifiable causes, (3) promising interventions and recommendations for future trials, and (4) possible biological mechanisms of fatigue. Cross-cutting themes were aphasia and the voice of people with lived experience. Working parties were formed and structured consensus building processes were followed.

**Results:**

We present 20 recommendations covering outcome measures for research, development, and testing of new interventions and priority areas for future research on the biology of post-stroke fatigue. We developed and recommend the use of the Stroke Fatigue Clinical Assessment Tool.

**Conclusions:**

By synthesizing current knowledge in post-stroke fatigue across clinical and pre-clinical fields, our work provides a roadmap for future research into post-stroke fatigue.

## Introduction

One in two stroke survivors experience post-stroke fatigue (pooled prevalence estimate 47% (95% CI = 43–50%)).^1^ Fatigue is a significant and disabling condition in its own right and is a significant barrier to engaging in rehabilitation and other activities that promote recovery. Despite its prevalence and impact, a recent systematic review of 200 stroke clinical guidelines found no strong recommendations for fatigue prevention or management.^2^ Fatigue is a critical unmet need which stroke survivors identify as a high-priority research area.^3^ Therefore, the International Stroke Recovery and Research Alliance selected post-stroke fatigue as a focus topic of their 3rd Stroke Recovery and Rehabilitation Roundtable (SRRR).

Post-stroke fatigue is not merely “tiredness,” nor simply physical deconditioning; some people have post-stroke fatigue despite high fitness levels.^4^ Post-stroke fatigue is not always associated with effort, nor always relieved by rest. Superficially, fatigue can seem like depression or apathy, and it may co-present with both, but is distinct. For the purposes of this work, we undertook a process of literature reviews, expert consensus, and engagement with people with lived experience of stroke to define to define post-stroke fatigue as:

. . . a feeling of exhaustion, weariness or lack of energy that can be overwhelming, and which can involve physical, emotional, cognitive and perceptual contributors, which is not relieved by rest and affects a person’s daily life.

Despite the high prevalence and burden of post-stroke fatigue, research is limited. Cohorts and assessment tools vary, making pooling data and systematic analysis difficult. Intervention trials are mostly underpowered and inconclusive. Few studies include participants with a speech-language disorder, and fewer have explored the relationship between fatigue and aphasia. Yet some hypothesize a bidirectional relationship^5^ given the large effort required to understand and/or produce language.

Clearly, post-stroke fatigue is a multi-faceted condition. An abundance of biopsychosocial factors are associated with fatigue,^6^ but causal relationships remain unclear, and there is overlap between depression and fatigue. Fatigue may predispose the development of depression, and fatigue can be a symptom of depression,^7^ but the selective efficacy of fluoxetine on depression and not fatigue^8^ suggests that they can be distinct. Fatigue likely hampers engagement in productive and meaningful activities, and elevates risk of social isolation and its secondary effects.

The overarching aim of this Roundtable was to bring together current knowledge of post-stroke fatigue based on best available evidence from multidisciplinary perspectives (clinical, pre-clinical, and lived experience), identify key knowledge gaps, and provide a roadmap for future research.

## Methods

**Figure 1.**
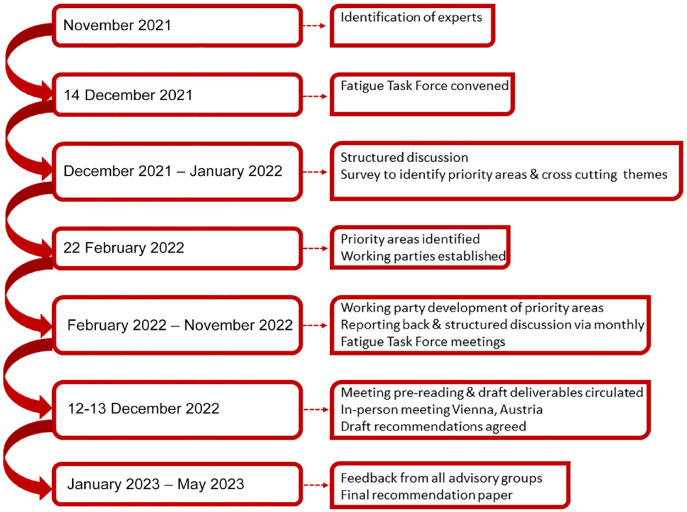
Fatigue task force process.

In December 2021, the Co-Chairs (C.E. and G.M.) identified international researchers (based on Scopus searches for most published authors in the field) to invite to the taskforce, following the principles of the SRRR initiative (diversity in discipline, geography, and gender). Through a process of structured discussion, rapid literature reviews, and surveys (Figure 1), the taskforce identified four priority questions for focus:

1. What is the best outcome measure of fatigue for research?
2. In clinical practice, how should fatigue and its potentially modifiable causes be identified?
3. What are the most promising interventions for post-stroke fatigue, and what are important considerations for future trials?
4. What are the possible biological mechanisms of fatigue?

Working parties for each question comprehensively investigated each topic and reported back to the taskforce for in-depth discussions (Supplemental documents describe membership). Cross-cutting task force themes were aphasia and the voice of people with lived experience. Our lived experience advisory group (LEAG; six people with post-stroke fatigue from four countries) reviewed and endorsed our priority questions, and were consulted regularly (Supplemental 1 provides a summary of involvement and feedback).

### What is the best available outcome measure of fatigue for research?

We aimed to reach consensus on an outcome measure for *research*. We took as a starting point a 2019 review^9^ that identified 24 different fatigue outcome measures consisting of 83 unique items, categorized into four main dimensions: (1) characteristics of fatigue, (2) severity, (3) fatigue interference, and (4) individuals’ management of fatigue. This review highlighted that no single tool measures all fatigue domains, there is little overlap between the domains measured by different tools, and there are none specifically designed for people with aphasia. Because none of the currently available outcome measures are ideal, we undertook a structured process (using Keeney’s^10^ Value Focussed Thinking methodology and graph-based theory voting) to reach consensus on the “best available” measure (see Supplemental 2 for full methods).

### How should fatigue be identified in clinical practice and potentially modifiable causes identified

Fatigue can be an invisible impairment, and acknowledgment of fatigue by clinicians can provide psychological relief.^11^ Therefore, we aimed to develop a clinical tool to ensure that (1) fatigue is identified and (2) potentially modifiable causes are identified. We took as a starting point known associations with post-stroke fatigue, the Greater Manchester Stroke Assessment Tool,^12^ and a review of 203 clinical practice guidelines.^2^ Through iterative discussions, the taskforce clarified the purpose of, and drafted the tool (Supplemental 3). Feedback was sought from 18 health professionals and our LEAG, and the tool refined.

### What are the most promising interventions for post-stroke fatigue management?

We considered interventions within a biopsychosocial framework. We use the term “treatment” to refer to interventions that aim to target the potential biology of fatigue (e.g. drugs, neuromodulation therapies). We use the term “management” for interventions aimed at the psychological and psychosocial aspects of fatigue (e.g. self-management, psychoeducation). Supplemental 4 describes the methods used to identify and synthesize the current evidence for both treatment and management interventions. In brief, we took systematic reviews published since 2015 as a starting point, set up alerts on Medline to identify new published trials, and searched clinical trial registries for relevant ongoing trials. We extracted and summarized (Table 1) identified randomized controlled trials (RCTs) in which fatigue was the primary outcome or intended target of the intervention. We also searched for interventions for fatigue management in conditions other than stroke (PubMed searched 8 July 2022 to retrieve systematic reviews and meta-analyses published from 2018 to 2022—see Supplemental Table 4.1 for search terms).

**Table 1.** Summary of trials of interventions for post-stroke fatigue where fatigue was the primary outcome or intended target of the intervention.

Author, country	n	Time since stroke	Intervention control	Outcome measure	Main findings
*Pharmacological treatments (including supplements and traditional Chinese Medicine)*					
**Bivard et al.**,^13^ **Australia**	**36**	**Late subacute to chronic**	**I: Modafinil** **C: Placebo**	**MFI**	**Significant difference between groups in favor of intervention (MD = −7.38, 95% CI = −21.76, −2.99)**
Poulsen et al.,^14^ Denmark	41	Acute	I: Modafinil C: Placebo	MFI-20	No significant difference between groups (underpowered primary endpoint noted)
Choi-Kwon et al.,^8^ South Korea	83	Late subacute to chronic	I: Fluoxetine C: Placebo	FSS VAS-f	No significant difference between groups (SMD = −0.38, 95% CI = −0.82, 0.05)
Johansson et al.,^15^ Sweden	6	Acute to chronic	I: Oral monoaminergic stabilizer C: Placebo	MFS	No significant difference between groups (SMD = −0.27, 95% CI = −1.99, 1.44)
Guo et al.,^16^ China	45	Acute to subacute	I: Citicoline C: Placebo	FSS	No significant difference between groups (SMD = −0.21, 95% CI = −0.83, 0.41)
**Gurak and Parfenov**,^17^ **Russia**	**30**	**Acute to early subacute**	**I: Thiamine (vitamin B12)** **+** **Standard care** **C: Standard care**	**MFI-20**	**Significant difference between groups in favor of intervention (SMD = **−**1.07, 95% CI = **−**1.85**, −**0.30)**
**Guo et al.**,^16^ **China**	**45**	**Acute to subacute**	**I: Traditional Chinese medicines** **C: Placebo**	**FSS**	**Significant difference between groups in favor of intervention (SMD = **−**4.35, 95% CI = **−**5.45**, −**3.22)**
**Liu et al.**,^18^ **Taiwan**	**64**	**Late subacute to chronic**	**I: ***Astragalus membranaceus***** **C: Placebo**	**BFI**	**Significant difference between groups in favor of intervention (MD = 9.8 (SD = 7.75), p** **=** **0.01)**
*Psychoeducational interventions*					
Mead et al.,^19^ United Kingdom	76	Late subacute to chronic	I: Education, information, goal setting, C: Information only	FAS	No significant differences between groups at 6 months (adjusted MD = −0.62, 95% CI = −4.96, 3.69)
**Nguyen et al.**,^20^ **Australia**	**15**	**NR**	**I: Cognitive behavioral therapy** **C: Standard care**	**FSS**	**Significant difference between groups in favor of intervention at 4** **months (MD = 1.92, 95% CI = 0.24, 3.60)**
Clarke et al.,^21^ New Zealand	19	Late subacute to chronic	Psychoeducation I: Targeting fatigue C: General education	FSS	No significant difference between groups (SMD = −0.10, 95% CI = −1.09, 0.89)
Zedlitz et al.,^22^ the Netherlands	83	Subacute to chronic	I: Cognitive therapy I: Cognitive therapy + graded exercise	CIS-f	No significant difference between groups (MD = 0.80, 95% CI = −3.63, 5.23)
*Neuromodulation interventions*					
**De Donker et al.**,^23^ **United Kingdom**	**30**	**Subacute to chronic**	**I: tDCS** **C: Sham tDCS**	**FSS-7** **VAS-f**	**Significant between group difference at 1** **week post intervention (W** **=** **52.5, Z** **=** **0.382, p** **=** **0.04)**
**Dong et al.**,^24^ **China**	**60**	**Late subacute to chronic**	**I: tDCS** **C: sham tDCS**	**FSS**	**Significant difference between groups in favor of intervention (at 4** **weeks)** **Intervention mean FSS score 32.1 (7.1)** **Control mean FSS score 37.2 (7.2)**
*Other interventions*					
**West et al.**,^25^ **Denmark**	**90**	**Acute to early subacute**	**Naturalistic lighting** **I: Artificial sunlight** **C: Standard indoor lighting**	**MFI**	**Significant difference between groups in favor of intervention at discharge (MD =–20.6%, 95% CI = –35.0%, –3.0%)**

MFI: Multidimensional Fatigue Inventory; CI: confidence interval; MFI-20: Multidimensional Fatigue Inventory-20; FSS: Fatigue Severity Scale; VAS-f: Visual Analogue Scale–fatigue; NR: not reported; CIS-f: Checklist Individual Strength–fatigue subscale; tDCS: transcranial direct current stimulation; FSS-7: Fatigue Severity Scale-7; MD: mean difference; SMD: standardised mean difference; MFS: Mental Fatigue Scale; BFI: Brief Fatigue Index.

Bolded studies report significant reductions in fatigue.

### What are the possible biological mechanisms of fatigue?

Understanding of potential underlying biological mechanisms for post-stroke fatigue may lead to improved treatments and management strategies. By a process of ranking, the mechanisms working group prioritized six topics for investigation (Supplemental 5). Targeted literature searches were conducted and key findings discussed within the mechanisms working party and taskforce.

## Results

Table 2 presents our recommendations for each priority question. The following section provides context and further details.

**Table 2.** Recommendations for future research in post-stroke fatigue.

Priority area	Recommendations
Measurement (research)	1. All studies of post-stroke fatigue should a. use the Fatigue Severity Scale-7 item (FSS-7) scale as the primary outcome measure. b. include simple visual analogue scales for fatigue severity and the impact of fatigue on communication ability. c. include qualitative evaluations where possible. d. include the interpretation and discussion of studies of post-stroke fatigue and explicitly consider the fatigue measure used and the domains that the measure covers (Figure 2).
Clinical assessment	2. Everyone who has had a stroke should be assessed for fatigue, using the Stroke Fatigue Clinical Assessment Tool administered by a health professional. When appropriate, referrals to other health professionals should be made.
Intervention development and testing	3. The most promising interventions for fatigue that should be prioritized for future research (alone and in combination) are a. psychoeducational interventions (including, but not limited to cognitive behavioral therapy) b. exercise and/or exercise memetics c. neuromodulation therapies d. dopamine re-uptake inhibitors.
	4. Development and testing of fatigue interventions should a. be based on a clearly described theoretical model of action. b. be developed in line with SRRR recommendations for intervention development and include people with lived experience in co-design. c. follow the SRRR trials decision-making framework to determine trial-readiness.
	5. Studies of new fatigue interventions should a. use the FSS-7 as the primary outcome measure. b. include relevant additional secondary measures aligned with the theoretical model of action. c. use the SRRRIII CONtrol deSIGN (CONSIGN) tool to design the most appropriate comparator. d. include people with aphasia and common co-morbidities including depression and anxiety.
Biological mechanisms	6. Priority areas for understanding the biology of post-stroke fatigue: a. inflammation and immune dysregulation, particularly the role of the mitochondria b. dopamine pathways, including the effect of dopamine re-uptake inhibitors and the mechanisms by which they act. c. neural network dysfunction d. brain imaging with precise delineation of lesions to better assess neuroanatomical associations

**Figure 2.**
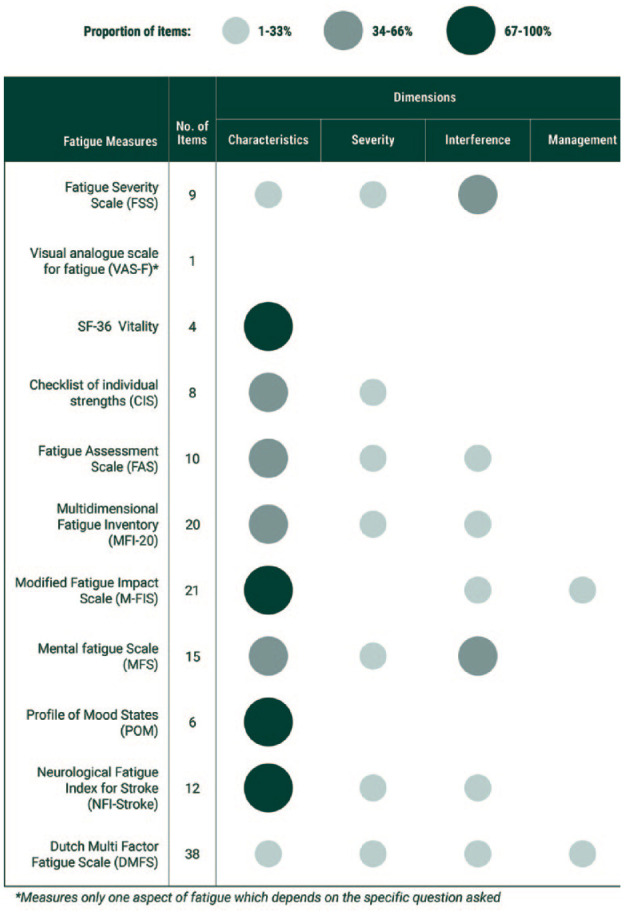
Domains of fatigue covered by outcome measures. Characteristics = perception of fatigue symptoms, including diurnal patterns; Severity = perceived intensity, including onset speed and recovery time; Interference = degree to which fatigue affects daily life; Management = degree to which coping strategies are used.^9^

### What is the best measure of fatigue for research?

The Fatigue Severity Scale (FSS)^26^ was the top-ranked measure against all of the desirable criteria, except one—“number of domains measured,” for which the Dutch Multifactor Fatigue Scale ranked highest (Supplemental 2 reports the scoring process in full). The FSS-7 is recommended (rather than the 9-item version) because the first two questions have poor item fit in Rasch analyses.^26^ Despite its name, the FSS mainly measures fatigue interference, not severity, and does not measure the impact of fatigue on communication ability. Therefore, the FSS-7 should be supplemented with simple visual analogue scales to measure these outcomes. Adaptation of the FSS-7 to make it accessible for people with language disorders has commenced. Until then, we recommend that people with aphasia receive communication support to complete the measure.

The minimum clinically important difference on the FSS-7 measure has not been established in stroke, and further work using anchor-based methods that relate changes on the FSS-7 scale to individual experiences is required. For now, it is reasonable to extrapolate minimum clinically important differences established for people with multiple sclerosis.^27^ Our work highlights that a single post-stroke fatigue score will not capture the impact of fatigue for an individual. Wherever possible, qualitative methods should be included to identify benefits that might not be otherwise captured.

The degree to which different fatigue outcome measures capture different domains of fatigue presents challenges for interpreting research findings. Figure 2 summarizes the domains of fatigue covered by the most commonly used measures. We recommend that research studies are interpreted in light of the domains covered. Nuanced interpretation of research findings using this framework will allow more accurate comparison between studies and may help reconcile conflicting research findings.

### Clinical assessment tool

Supplemental 3 summarizes the evidence for factors associated with post-stroke fatigue. The Stroke Fatigue Clinical Assessment Tool (SF-CAT) is presented in Table 3. The SF-CAT is designed to be administered via interview with a health professional. We recommend its use as part of comprehensive assessment for all survivors of stroke.

**Table 3.** Stroke Fatigue Clinical Assessment Tool (SF-CAT). The purpose of the tool is twofold; to ensure (1) fatigue is not missed as an unmet need *(simple acknowledgment of fatigue as an issue for people with stroke can greatly reduce distress)* and (2) potentially modifiable causes are identified. It is designed for any health care professional to administer via interview, using the questions in each of the categories. Modifiable factors should be addressed where possible, with referral to other health professionals as needed.

Ask your patient/client if they:	
*Assess whether fatigue is an issue*	
Feel tired all the time or get tired quickly since your stroke? Need additional help and support for this?	Y: screen for the potential causes and precipitating factors (below), use FSS-7 for quantitative assessment
*Consider mood disorders*	
Feel sad or depressed? Feel anxious or stressed?	Y: screen for depression (e.g. PHQ9) Y: screen for anxiety (e.g. GAD7)
*Consider sleep quality*	
Have difficulty falling or staying asleep? Wake up frequently, or wake feeling unrefreshed? Fall asleep unintentionally during the day?	Y: screen for insomnia, depression, and/or anxiety Y: screen for sleep apnea/other sleep disorders (e.g. GSAQ)
*Consider new/uncontrolled conditions*	
Have any new pain that bothers you? Have hypotension? Have chronic conditions (diabetes, hypothyroidism, anemia, etc.) that are not optimally controlled?	Y: assess pain Y: address/refer Y: address/refer
*Consider physical/nutrition status*	
Exercise regularly? Keep active? Regularly miss meals?	N: address/refer Y: address/refer
*Consider role of medication*	
Get side effects from your medications (e.g. beta blockers, benzodiazepines, polypharmacy)?	Y: address/refer
Drink alcohol?	Y: how much and how often? address/refer
*Consider new/undiagnosed cognitive impairment*	
Have new problems remembering things or concentrating?	Y: screen for cognitive impairment (e.g. MoCA)
*Consider speech and/or language disorder*	
Do you often feel fatigued after talking or listening to others talk?	Y: Screen (e.g. sections 9 and 10 NIH Stroke Scale) refer as appropriate

FSS-7: Fatigue Severity Scale; PHQ9: Patient Health Questionnaire; GAD7: Generalized Anxiety Disorder; GSAQ: Global Sleep Assessment Questionnaire; MoCA: Montreal Cognition Assessment; NIH: National Institute of Health Stroke Severity Scale.

### What are the most promising interventions for post-stroke fatigue

We identified five systematic reviews published since 2015 exploring the effect of interventions for post-stroke fatigue. Not all reviews restricted inclusion to trials where fatigue was the primary outcome or intended intervention target. From these reviews, and additional search methods (Supplemental 4), we identified and extracted data for 15 unique randomized trials (Table 1). Although some small trials reported benefits from Modafinil, psychoeducational interventions, and neuromodulation therapies, there are conflicting results from other trials. Our search of the World Health Organization Clinical Trial Register identified a number of, mostly small, ongoing or unpublished trials (Supplemental Table 4.4).

Unsurprisingly, no clinical guidelines make strong recommendations about post-stroke fatigue management (Supplemental Table 4.3). Recently updated guidelines from Canada,^28^ Australia and New Zealand,^29^ the United Kingdom and Ireland,^30^ and the American Heart Association scientific statement^31^ provide consensus-based suggestions for education, exercise, and energy conservation, identifying potential modifiable factors and sleep hygiene.

With regard to interventions for fatigue management in conditions other than stroke (Supplemental Table 4.5), education, cognitive behavioral therapy, and exercise were commonly reported as effective (albeit with low certainty) for a range of conditions. In neurological conditions, exercise likely improves fatigue,^32^ although not all systematic reviews report significant findings.

People with aphasia have been excluded from most previous fatigue intervention trials.^5^ Clinicians with experience treating communication disorders frequently employ fatigue management strategies during treatment sessions (e.g. taking rest or exercise breaks, pacing);^33^ these strategies warrant testing in clinical trials.

Future clinical trials of fatigue management should carefully consider issues, including participant selection and combined intervention development and study design. For participant selection, either the validated case definition for fatigue^34^ (noting that this may miss some fatigue cases)^35^ or the Greater Manchester Screening tool^12^ should be used. Symptoms of depression and anxiety should generally not be exclusion criteria and should be assessed at baseline and follow-up. Trial materials and interventions must be accessible for people with communication disorders.

New fatigue interventions should be co-designed by people with lived experience of fatigue, clinicians, and multidisciplinary researchers. Designs should include elucidation of a theoretical model underpinning the hypothesized mechanism of action. Models need not focus on biological mechanisms—for example, interventions aiming to improve self-management may be based on an assumption around improving resilience and coping strategies or reducing anxiety. Theoretical models that reflect mediating/moderating relationships should guide the choice of outcome measures and overall trial design. Studies of fatigue interventions should include FSS-7 as the primary outcome measure. Choice of secondary measures should be hypothesis driven and align to the underpinning theoretical model. For example, if the hypothesized mechanism of the intervention is to reduce fatigue via anxiety reduction, then measures of anxiety should be included.

Future trials of interventions for post-stroke fatigue require careful development. The SRRR trials development framework (and supplemental flow charts) should be used to determine when an intervention is ready for testing in a definitive randomized trial.^36^ Key knowledge units are important in making GO/NO-GO decisions, including WHO, HOW MUCH, and WHEN. Alternative study designs, including dose-finding studies, single case experimental designs, and hypothesis-specific pilot trials, can help build necessary knowledge units. Future randomized trials should include careful development of the control intervention^37^ and ideally include measures of change at a biological level.

### What are the possible biological mechanisms of post-stroke fatigue

The mechanisms of post-stroke fatigue are presently unknown. Based on studies of fatigue in stroke and other related conditions, we identified promising directions for future research into likely mechanisms.

Many small studies have investigated the relationship between lesion characteristics and fatigue with inconclusive results partly due to variations in fatigue measures, imaging methods, and time points post-stroke. In a recent meta-analysis,^6^ lesions in the thalamus were associated with greater likelihood of fatigue (odds ratio (OR) = 1.76 (1.09, 2.85)) in people with chronic stroke, but no associations between hemisphere of stroke or cortical versus subcortical lesions were found. A recent large (n = 361) study,^38^ using diffusion-weighted magnetic resonance imaging, also found significant associations between thalamic lesions and the likelihood (OR = 2.67 (1.46, 4.88)) and severity of fatigue at 6 months post-stroke. More sensitive brain imaging methods and precise delineation of lesions are needed to confirm neuroanatomical associations with fatigue.^39^

Systemic inflammation may contribute to post-stroke fatigue. Several studies have identified inflammatory biomarkers in the acute phase of stroke that may be associated with later fatigue. A retrospective medical record audit (n = 178)^40^ found significantly elevated erythrocyte sedimentation rate in people with post-stroke fatigue compared to people post-stroke without fatigue. In a prospective study^41^ (n = 333), elevated serum neutrophil-to-lymphocyte ratio was independently associated with fatigue at 6 months (OR = 11.13 (4.64, 26.70)), as was self-rated depression (OR = 1.13 (1.03, 1.23)). Other markers of systemic inflammation, including inflammatory cytokines (e.g. Interleukin-1, C-reactive protein), are associated with fatigue.^42^ Excessive cytokine production and immune dysregulation decrease several neurotransmitters, which could play a role.^43^ Following stroke, microglia (the resident immune cells of the brain) become overactive, driving chronic neuroinflammation.^44^ Links between microglial activation and post-stroke fatigue are yet to be directly investigated. While a few small studies have investigated the effect of anti-inflammatory agents on post-stroke fatigue,^45,46^ further definitive trials are needed.

There are several other inflammatory processes that may contribute to fatigue in other diseases that have not been examined in stroke. Genetics, including single-nucleotide polymorphisms that modulate inflammation, could play a role but few studies have investigated this.^47^ Gut dysbiosis is associated with immune and inflammatory responses following stroke in animals/humans^48^ and may play a role in chronic fatigue syndrome.^49^ Gut dysbiosis warrants exploration as a potential treatment target for post-stroke fatigue via dietary interventions. Similarly, thyroid-stimulating hormone serum levels are inversely associated with fatigue acutely (OR = 0.30 (0.24, 0.37)) and at 6 months post-stroke (OR = 0.70 (0.58, 0.84)).^50^ Given the known association between hypothyroidism and fatigue,^51^ it is possible that decreased thyroid-stimulating hormone contributes to post-stroke fatigue.

Post-stroke alteration of cellular energy stores could also play a role. Systemic inflammation can disturb mitochondrial function shifting it toward a more inefficient form of metabolism: a phenomenon observed in cancer and chronic fatigue syndrome.^43^ Stroke-induced inflammation may lead to genomic or epigenetic changes which modulate brain energy metabolism.^52^ Compensatory recruitment of undamaged neural circuitry after stroke is a key neuroplastic response contributing to post-stroke recovery.^53^ As a result, engaging in different tasks could increase demands on cellular energy stores, thereby contributing to fatigue. Further research on the effects of metabolic load and post-stroke fatigue is warranted.

Dysfunction in sensorimotor processing may contribute to fatigue after stroke.^54^ People with post-stroke fatigue have reduced motor cortical excitability, and an imbalance in inter-hemispheric inhibition, the effect of which could contribute to fatigue.^55^ Early research suggests a possible relationship between fatigue and resting-state hyper-connectivity in sensory networks and hypo-connectivity in motor networks.^56^ Two small studies have found reductions in fatigue following neuromodulation via non-invasive brain stimulation^23,24^. Neural network dysfunction is a possible mechanism and intervention target; several trials are ongoing (Supplemental Table 4.4).

Dopamine neurons regulate movement, motivation, arousal, and the immune system. Damage to dopamine neurons is associated with fatigue in other diseases.^57^ Dopamine re-uptake inhibitor drugs (e.g. Modafinil) have shown some effectiveness^13^ in reducing post-stroke fatigue. Clinical trials that use imaging techniques to relate behavioral outcomes to temporal changes in functional network connectivity, including dopamine activity, are required to elucidate the underlying mechanisms of pharmacological treatments on fatigue.

Exercise could target multiple potential biological mechanisms, including inflammation and dopamine. Exercise improves aerobic conditioning, reduces mild to moderate depression and anxiety, improves sleep quality, increases dopamine levels, elevates central nervous system growth factors, and decreases inflammation.^32,58^ Surprisingly, only one RCT has tested the effect of exercise (in combination with cognitive therapy); several RCTs are ongoing (Supplemental Tables 4.1 and 4.4). Exercise mimetics (drugs that activate similar cellular signaling pathways as aerobic exercise) warrant investigation.^59^

## Discussion

The research field of post-stroke fatigue is in its infancy, having evolved largely in disciplinary silos where progress has been hampered by inconsistencies in definitions, measurement tools, and terminology. Our group has developed consensus in definition, clinical screening tools, and outcome measurement. By highlighting the complex nature of fatigue, and the lack of consistency in the domains of fatigue covered by current outcome measurement tools, we have provided a framework to support better interpretation of research findings. Our clinical screening tool will support clinicians to identify fatigue and to consider potentially modifiable contributing factors.

A number of questions remain unanswered, including whether or not distinct sub-types of post-stroke fatigue exist and/or are important. Observational studies suggest the existence of early- and late-onset fatigue,^60^ although superficially, the description of the experience of fatigue from stroke survivors is remarkably similar.^11^ At a self-management level, effective interventions may be similar, regardless of the underlying biology. On the contrary, future work may elucidate differences in underlying causative mechanisms for fatigue that could lead to different treatment targets.

Understanding the biological mechanisms of post-stroke fatigue is key to developing new and effective therapeutic interventions. There may be several different underlying mechanisms at play at an individual level, and/or mechanisms may be different between individuals. Further work is needed to understand the underlying biology and to test possible treatments. In the reverse direction, understanding how some treatments, for example, Modafinil, work at a biological level will assist in understanding why some people respond and others do not. While we know a range of biopsychosocial factors are associated with post-stroke fatigue, causative relationships remain unclear.^6^ However, biopsychosocial frameworks may be useful to consider when designing combination interventions (e.g. exercise and cognitive behavioral therapy) for fatigue.

There is currently very limited evidence for any interventions to treat or manage fatigue. With that caveat, psychoeducational interventions, exercise, dopamine re-uptake inhibitors, and neuromodulation therapies warrant further investigation. Combination therapies may be more effective for fatigue than single interventions given the lessons learned from stroke recovery trials which show that combination therapies are more effective than single treatment targets.^61,62^ Not all interventions will be acceptable (e.g. exercise) or accessible (e.g. neuromodulation therapies) to everyone with post-stroke fatigue, therefore a range of interventions should be investigated. Intervention studies could consider incorporating a run-in period during which potential contributing factors for fatigue are identified (using the SF-CAT tool) and optimally managed before specific fatigue interventions are tested. Given the amount of conflicting positive and negative trial results, and limited attempts to understand and explain the discrepancies, it is vital that future trials are carefully and thoughtfully designed. We have provided recommendations about how to do this.

Our work has a number of key strengths. First, we involved a diverse group of international experts from a range of clinical and pre-clinical disciplines and included recognized experts from related fields who worked together for >18 months to review relevant literature and discuss this in depth. Second, we worked closely with a group of individuals living with post-stroke fatigue, who provided feedback to the taskforce at each step of the process. Third, we sought to tackle the key gaps in understanding fatigue. The limitation of this approach made it problematic to condense the key messages into one paper; therefore, readers are encouraged to refer to the supplemental materials. Future papers from this task force will expand on the findings. Finally, we followed an established structured formalized process of ranking for our measurement consensus process. Our work has some limitations. Due to restrictions in time and resources, we did not conduct rigorous systematic reviews, and thus, some studies may have been missed although the diversity and expertise within our group mitigates this risk. The Stroke Fatigue Clinical Assessment Tool does not include specific recommendations or pathways to address each potentially contributing factor—this was beyond the scope of our work.

## Conclusion

As experts from a range of disciplines, we have synthesized current knowledge in post-stroke fatigue across clinical and pre-clinical fields and provided a roadmap for future research. We believe this will lead to greater investment in fatigue research which will produce major breakthroughs and ultimately improve the lives of people living with fatigue after stroke.

## Supplemental Material

sj-docx-1-wso-10.1177_17474930231189135 – Supplemental material for A roadmap for research in post-stroke fatigue: Consensus-based core recommendations from the third Stroke Recovery and Rehabilitation Roundtable Click here for additional data file. Supplemental material, sj-docx-1-wso-10.1177_17474930231189135 for A roadmap for research in post-stroke fatigue: Consensus-based core recommendations from the third Stroke Recovery and Rehabilitation Roundtable by Coralie English, Dawn B Simpson, Sandra A Billinger, Leonid Churilov, Kirsten G Coupland, Avril Drummond, Annapoorna Kuppuswamy, Mansur A Kutlubaev, Anners Lerdal, Amreen Mahmood, G Lorimer Moseley, Quentin J Pittman, Ellyn A Riley, Brad A Sutherland, Connie HY Wong, Dale Corbett and Gillian Mead in International Journal of Stroke

sj-docx-2-wso-10.1177_17474930231189135 – Supplemental material for A roadmap for research in post-stroke fatigue: Consensus-based core recommendations from the third Stroke Recovery and Rehabilitation Roundtable Click here for additional data file. Supplemental material, sj-docx-2-wso-10.1177_17474930231189135 for A roadmap for research in post-stroke fatigue: Consensus-based core recommendations from the third Stroke Recovery and Rehabilitation Roundtable by Coralie English, Dawn B Simpson, Sandra A Billinger, Leonid Churilov, Kirsten G Coupland, Avril Drummond, Annapoorna Kuppuswamy, Mansur A Kutlubaev, Anners Lerdal, Amreen Mahmood, G Lorimer Moseley, Quentin J Pittman, Ellyn A Riley, Brad A Sutherland, Connie HY Wong, Dale Corbett and Gillian Mead in International Journal of Stroke

sj-docx-3-wso-10.1177_17474930231189135 – Supplemental material for A roadmap for research in post-stroke fatigue: Consensus-based core recommendations from the third Stroke Recovery and Rehabilitation Roundtable Click here for additional data file. Supplemental material, sj-docx-3-wso-10.1177_17474930231189135 for A roadmap for research in post-stroke fatigue: Consensus-based core recommendations from the third Stroke Recovery and Rehabilitation Roundtable by Coralie English, Dawn B Simpson, Sandra A Billinger, Leonid Churilov, Kirsten G Coupland, Avril Drummond, Annapoorna Kuppuswamy, Mansur A Kutlubaev, Anners Lerdal, Amreen Mahmood, G Lorimer Moseley, Quentin J Pittman, Ellyn A Riley, Brad A Sutherland, Connie HY Wong, Dale Corbett and Gillian Mead in International Journal of Stroke

sj-docx-4-wso-10.1177_17474930231189135 – Supplemental material for A roadmap for research in post-stroke fatigue: Consensus-based core recommendations from the third Stroke Recovery and Rehabilitation Roundtable Click here for additional data file. Supplemental material, sj-docx-4-wso-10.1177_17474930231189135 for A roadmap for research in post-stroke fatigue: Consensus-based core recommendations from the third Stroke Recovery and Rehabilitation Roundtable by Coralie English, Dawn B Simpson, Sandra A Billinger, Leonid Churilov, Kirsten G Coupland, Avril Drummond, Annapoorna Kuppuswamy, Mansur A Kutlubaev, Anners Lerdal, Amreen Mahmood, G Lorimer Moseley, Quentin J Pittman, Ellyn A Riley, Brad A Sutherland, Connie HY Wong, Dale Corbett and Gillian Mead in International Journal of Stroke

sj-docx-5-wso-10.1177_17474930231189135 – Supplemental material for A roadmap for research in post-stroke fatigue: Consensus-based core recommendations from the third Stroke Recovery and Rehabilitation Roundtable Click here for additional data file. Supplemental material, sj-docx-5-wso-10.1177_17474930231189135 for A roadmap for research in post-stroke fatigue: Consensus-based core recommendations from the third Stroke Recovery and Rehabilitation Roundtable by Coralie English, Dawn B Simpson, Sandra A Billinger, Leonid Churilov, Kirsten G Coupland, Avril Drummond, Annapoorna Kuppuswamy, Mansur A Kutlubaev, Anners Lerdal, Amreen Mahmood, G Lorimer Moseley, Quentin J Pittman, Ellyn A Riley, Brad A Sutherland, Connie HY Wong, Dale Corbett and Gillian Mead in International Journal of Stroke

sj-pptx-6-wso-10.1177_17474930231189135 – Supplemental material for A roadmap for research in post-stroke fatigue: Consensus-based core recommendations from the third Stroke Recovery and Rehabilitation Roundtable Click here for additional data file. Supplemental material, sj-pptx-6-wso-10.1177_17474930231189135 for A roadmap for research in post-stroke fatigue: Consensus-based core recommendations from the third Stroke Recovery and Rehabilitation Roundtable by Coralie English, Dawn B Simpson, Sandra A Billinger, Leonid Churilov, Kirsten G Coupland, Avril Drummond, Annapoorna Kuppuswamy, Mansur A Kutlubaev, Anners Lerdal, Amreen Mahmood, G Lorimer Moseley, Quentin J Pittman, Ellyn A Riley, Brad A Sutherland, Connie HY Wong, Dale Corbett and Gillian Mead in International Journal of Stroke
