# Supplementary material for: A roadmap for research in post-stroke fatigue: Consensus-based core recommendations from the third Stroke Recovery and Rehabilitation Roundtable
Source: Int J Stroke. 2023 Oct 12;19(2):133–44. doi: 10.1177/17474930231189135 (PMC10811972; doi:10.1177/17474930231189135)
Supplement: sj-docx-1-wso-10.1177_17474930231189135 – Supplemental material for A roadmap for research in post-stroke fatigue: Consensus-based core recommendations from the third Stroke Recovery and Rehabilitation Roundtable [file sj-docx-1-wso-10.1177_17474930231189135.docx]

**Supplemental 1**

**Methods for engaging with the Lived-experience Advisory Group (LEAG)**

Members of the Fatigue Task Force identified survivors of stroke in their local networks who might be interested in providing feedback for the Stroke Recovery and Rehabilitation Roundtable process. We contacted 6 survivors of stroke from 4 countries (Australia, United Kingdom, USA, India) who all agreed to participate. Three members of the group lived with aphasia, 4 were female, age ranged from 40-75 years.

Four rounds of feedback were sought. In each round, a member of the Fatigue Task Force (Dawn Simpson) met individually with each member via video conference to discuss feedback on key questions.

**Feedback round 1**: We asked the LEAG for their feedback on the key Fatigue Task Force topics that were identified: definition of fatigue; measurement of fatigue; understanding of the causes of fatigue. The specific questions asked, and the combined summary of responses is shown in Table 1.1 below.

**Feedback round 2**: We asked the LEAG for their feedback on other medical issues they noted had mimicked their post-stroke fatigue (differentiation / development of clinical tool topic) and about any successful interventions for their post-stroke fatigue that had be beneficial for them.

**Feedback round 3**: We asked the LEAG for their feedback on a draft version of the clinical tool, specifically were there any factors from their experience that we had not captured in the tool.

A member of the LEAG virtually attended the Stroke Recovery and Rehabilitation Roundtable in-person meeting (December 2022) and provided feedback on the summary meeting discussions.

**Feedback round 4**: The LEAG provided feedback about the proposed key recommendations for the Fatigue Task Force.

**Table 1.1 Consumer feedback on Fatigue Task Force priority topics identified by the Core Group Members**

| Topic areas | **Definition** | **Measurement** | **Causes/mechanisms** |
| --- | --- | --- | --- |
| Questions | **Fatigue Task Force working definition for post-stroke fatigue:**  **Does this match your experience? Is anything missing?** | **As a stroke survivor, what is it about your post-stroke fatigue that you think should be measured? e.g.: Ways it affects your life, how it feels, your level of coping with it, how severe it is, is a score (knowing change) helpful?** | **For you, is it important that you understand the causes of post-stroke fatigue and why? Is it helpful for education about fatigue?** |
| Summary comments | Yes (all 6 CAG members)  Varies from sudden onset to building lethargy through the day. Anywhere, anytime Definitely persisting. Both physical and cognitive, not always relieved by rest or sleep Emotionally draining Clarity of definition important for stroke survivors and others (health professionals and support people) Need to know what it is, and what it is not Fatigue as a word is a complication and problematic – wrong word. People make assumptions i.e. you are just tired BUT thinking is hard, eating is hard, everything is hard. | Important that a measure captures: Ways fatigue affects your life (including personality); how severe it is; how it feels; how you are coping  Score important. Useful for research but *“getting better is thrilling!”* Complex experience **-** important to capture all the ways it’s affecting you “*It’s not an amoeba, it’s a complex organism!*” Small changes important for 3^rd^ parties (researchers). Big changes important for stroke survivors Positive value as an assessment to check progress but care needed if fatigue is not changing, measuring may not be good for your mood | If understand the cause– maybe at an individual level can do something about it Need to know the target (for treatment) Mechanisms important as intervention has implications for stroke survivor. E.g. medication is costly - stroke survivors want to know that it is effectively treating the right thing |
