# Supplementary material for: A roadmap for research in post-stroke fatigue: Consensus-based core recommendations from the third Stroke Recovery and Rehabilitation Roundtable
Source: Int J Stroke. 2023 Oct 12;19(2):133–44. doi: 10.1177/17474930231189135 (PMC10811972; doi:10.1177/17474930231189135)
Supplement: sj-docx-2-wso-10.1177_17474930231189135 – Supplemental material for A roadmap for research in post-stroke fatigue: Consensus-based core recommendations from the third Stroke Recovery and Rehabilitation Roundtable [file sj-docx-2-wso-10.1177_17474930231189135.docx]

**Supplemental 2**

**Methods for reaching consensus for recommended outcome measure.**

Members of the working party for measurement of fatigue were Anners Lerdal, Coralie English, Leonid Churilov, Gillian Mead, Ellyn Riley, Avril Drummond, Dawn Simpson, Amreen Mahmood.

We acknowledge and thank the following individuals for their contribution to the consensus process: Suzanne Barker-Collo, Natasha Lannin, Kirsten Coupland.

Our consensus building process followed the same methodology as previous SRRR work. (1) We took as our starting point the 2019 Skogestad et al. review (2) of outcome measures of fatigue used in stroke research. The review results identified 78 studies, 24 different measures of fatigue consisting of 156 different items (questions). The authors of the review conducted a qualitative analysis, identifying 83 unique items that could be categorized into 4 main dimensions of fatigue: 1) characteristics of fatigue, 2) severity, 3) fatigue interference and 4) individuals’ management of fatigue.

We first short listed the outcome measures to take forward to consensus. We based our decision on the following factors (i) how frequently it has been used in studies of post-stroke fatigue to date, (ii) whether it has basic face validity (e.g. we excluded SF-36 vitality scale), (iii) measures >1 dimensions of fatigue (i.e. we excluded Visual Analogue Scale for fatigue) and (iv) whether it was specifically developed or adapted to post-stroke fatigue. Our short-listed outcome measures were:

- - Fatigue Severity Scale (FSS)
  - Fatigue Assessment Scale (FAS)
  - Multidimensional Fatigue Inventory (MFI-20)
  - Modified Fatigue Impact Scale (M-FIS)
  - Neurological Fatigue Index for Stroke (NFI-Stroke)
  - Dutch Multifactor Fatigue Scale (DMFS)

Aligned with previous SRRR work, we used Keeney’s Value Focussed Thinking methodology (3) to elicit a list of 9 desirable criteria that should be included in a ‘good’ outcome measure (Supplementary Table 2.1).

**Table 2.1 Desirable criteria for a ‘good’ outcome measure of fatigue**

| **Frequency of use of the tool in previous research**  (e.g. how familiar are researchers with the tool and therefore more likely to go with what they know) |
| --- |
| **Number of domains of fatigue the tool captures**  (Based on the work of Skogestad 2019 ([https://doi.org/10.1016/j.jpsychores.2019.109759](https://doi-org.ezproxy.newcastle.edu.au/10.1016/j.jpsychores.2019.109759)) e.g. up to 4 domains across *characteristics; severity; interference; coping*) |
| **Face validity for stroke**  (e.g. stroke specific, accounts for impact of other stroke sequelae on fatigue, assesses presence of pre-stroke fatigue) |
| **Construct validity**  (e.g. established validity in stroke/other conditions – i.e. psychometric properties, able to differentiate fatigue from other mimics, e.g. daytime sleepiness, depression, apathy) |
| **Responsiveness and sensitivity:**  (e.g. established minimal clinically important difference, responsiveness to clinically meaningful change, discriminative (how many participants are correctly classified), low floor and ceiling effects, Rasch analysis of good item fit) |
| **Reliability**  (e.g. inter- and intra-rater, test-retest, established measurement error) |
| **Feasibility**:  (easy to administer, short time to administer) |
| **Accessibility**  (e.g. no cost, optimised for or validated in populations with aphasia, translated to multiple languages) |
| **Ability to run meaningful statistical analysis:**  (e.g. ordinal vs continuous measures, established measurement error, easy to interpret scores, validated cut off score) |

Task force members and 3 additional researchers (n=13 total) ranked (i) the desirable criteria in order of importance and (ii) how well each outcome measure performs against each criterion. To support decision making during ranking, we searched PubMed for studies on measurement properties using a search filter developed by the Consensus-based Standards for the selection of health Measurement Instruments group (4) and extracted key information regarding psychometric properties of the short-listed outcome measures. We used graph-based theory voting (5) system to synthesise the responses. Ranked criteria are in Supplemental Table 2.2 and the results of the graph-based theory voting are in Supplemental Table 2.3.

**Table 2.2 Desirable criteria ranked by importance**.

| Key characteristic in ranked order | Ranking (1 highest, **bold = tied** ranking) |
| --- | --- |
| Construct validity | 1 |
| Responsiveness | 2 |
| **Number of dimensions measured** | **3** |
| **Face validity** | **3** |
| Reliability | 4 |
| Feasibility | 5 |
| Accessibility | 6 |
| Ability to run meaningful statistical analyses | 7 |
| Frequency of use in prior research | 8 |

**Table 2.3 Results of graph-based theory voting for performance of outcome measures against desirable criteria.**

| Characteristic in ranked order (**bold = tied** ranking) | top rated measure | tied with |
| --- | --- | --- |
| Construct validity | FSS |  |
| Responsiveness | FSS | MFI-20 |
| **Number of domains measured** | DMFS |  |
| **Face validity** | FSS |  |
| Reliability | FSS |  |
| Feasibility | FSS |  |
| Accessibility | FSS |  |
| Ability to run meaningful statistical analyses | FSS | mFIS and MFI-20 |
| Frequency of use in prior research | FSS |  |

FSS = Fatigue Severity Scale. DMFS = Dutch Multifactor Fatigue Scale; MFI-20: Multidimensional Fatigue Index; mFIS: modified Fatigue Impact Scale

**References**

1. Kwakkel G, Lannin NA, Borschmann K, English C, Ali M, Churilov L, et al. Standardized measurement of sensorimotor recovery in stroke trials: consensus-based core recommendations from the stroke recovery and rehabilitation roundtable. Neurorehabilitation and neural repair. 2017;31(9):784-92.

2. Skogestad IJ, Kirkevold M, Indredavik B, Gay CL, Lerdal A. Lack of content overlap and essential dimensions–A review of measures used for post-stroke fatigue. Journal of Psychosomatic Research. 2019;124:109759.

3. Keeney RL. Value-focused thinking: Identifying decision opportunities and creating alternatives. European Journal of operational research. 1996;92(3):537-49.

4. Mokkink LB, Terwee CB, Patrick DL, Alonso J, Stratford PW, Knol DL, et al. The COSMIN checklist for assessing the methodological quality of studies on measurement properties of health status measurement instruments: an international Delphi study. Quality of life research. 2010;19:539-49.

5. Utley M, Gallivan S, Mills M, Mason M, Hargraves C. A consensus process for identifying a prioritised list of study questions. Health care management science. 2007;10(1).
