# Supplementary material for: A roadmap for research in post-stroke fatigue: Consensus-based core recommendations from the third Stroke Recovery and Rehabilitation Roundtable
Source: Int J Stroke. 2023 Oct 12;19(2):133–44. doi: 10.1177/17474930231189135 (PMC10811972; doi:10.1177/17474930231189135)
Supplement: sj-docx-3-wso-10.1177_17474930231189135 – Supplemental material for A roadmap for research in post-stroke fatigue: Consensus-based core recommendations from the third Stroke Recovery and Rehabilitation Roundtable [file sj-docx-3-wso-10.1177_17474930231189135.docx]

**Supplemental 3**

**Summary of evidence underpinning the development of the Stroke Fatigue Clinical Assessment Tool (SF-CAT)**

Members of the working party for the clinical assessment of fatigue were Coralie English, Mansur Kutlubaev, Dawn Simpson.

**Declaration of funding:** Mansur Kutlubaev was supported by the Bashkir State Medical University Strategic Academic Leadership Program (PRIORITY-2030)

Fatigue after stroke should be differentiated from other conditions that can mimic or contribute to the experience of fatigue. Only two clinical practice guidelines include recommendations about fatigue management providing consensus-based recommendations about screening for potentially modifiable factors:

*“Avoiding sedating drugs and alcohol, and screening for sleep-related breathing disorders and depression”*, (1) and

*“screened for common and treatable poststroke co-morbidities and for medications that are associated with and/or exacerbate fatigue (Evidence Level B), including a. signs of depression or other mood-related conditions b. sleep disorders or factors that decrease quality of sleep (e.g. sleep apnea, pain), and c. other common poststroke medical conditions and medications that increase fatigue (e.g. systemic infection such as UTI, dehydration, sedating drugs, thyroid disorders, or other general medical problems).”* (2)

Several factors have been associated with greater likelihood of developing fatigue (see Supplementary Table 3.1). Importantly, we do not know if any of these factors are causative. They include demographic, psychological, physiological, clinical (neurological), neuroimaging and other parameters. The quality of evidence behind these factors varies greatly. Zhang et al. performed systematic review and meta-analysis which aimed to identify factors associated with post-stroke fatigue. (3) Female sex (OR = 1.39; p < 0.01), thalamic lesions (OR= 1.76; p = 0.02), leucoaraiosis (OR = 1.73; p < 0.01), NIHSS score (OR = 1.16; p < 0.01), modified Rankin Scale (OR = 1.63; p < 0.01), depression (OR = 1.75; p < 0.01), sleeping disturbances (OR = 2.01; p < 0.01), and diabetes mellitus (OR = 1.47; p = 0.05) were significantly associated with post-stroke fatigue. Depression (OR = 1.46; p < 0.01), anxiety (OR = 1.13; p < 0.01) and sleeping disturbances (OR = 1.98; p < 0.01) were related to fatigue presence at more than 6 months after stroke.

Other factors that could be potentially related to post-stroke fatigue are pain and social factors (social support, social utilization). Single studies showed that certain psychological factors (locus of control, copying style), (4) inflammatory markers (cytokine levels), (5) medications (statins, antidepressants, polypharmacy), and autonomic dysfunction (arterial hypo- or hypertension) (6) may be associated to the development of post-stroke fatigue. (7)

**Table 3.1 Factors associated with the development of post-stroke fatigue.**

| **Groups of factors** | **Factors** |
| --- | --- |
| Socio-demographic factors | **Female gender**, social support, social utilization |
| Psychological factors | **Depression, anxiety**, coping style (avoidance; confrontational), locus of control, cognitive decline, physical fitness |
| Physiological/metabolic factors | **Sleep disorders,** **diabetes mellitus,** hypo- or hypertension; cardiac arrhythmia, musculoskeletal and post-stroke pain. |
| Neurological factors | **NIHSS score, modified Rankin Scale** |
| Neuroimaging factors | **Thalamus, leucoaraiosis, infratentorial strokes,** basal ganglia |
| Laboratory factors | Cytokines, thyroid stimulating hormone |
| Pharmacological factors | Antidepressants, statins, polypharmacy |
| Genetic factors | Polymorphisms of genes associated with inflammatory response |

Bolded factors are those that were found to have significant associations with post-stroke fatigue in the recent meta-analysis, (3) the others were identified in other studies, and either were not included or were not significant in the meta-analysis. (7)

Some of these factors warrant further definition and discussion.

Depression after stroke usually manifests with symptoms which could be divided into three categories: *emotional* (low mood, hopelessness, and anhedonia), *cognitive* (ideas of guilt, failure, worthlessness, and self-harm) and *behavioral* (loss of appetite, disorders of concentration, sleep disorders, psychomotor changes and *fatigue*). (8) The relationship between fatigue and depression is bidirectional; fatigue predisposes the development of depression and depression may manifest with symptoms of fatigue. The association between these two phenomena remains stable at 6 months’ follow-up according to one study. (9) However, fatigue and depression are different phenomena. For instance, there is evidence from one small trial that antidepressant fluoxetine improves depression but does not affect fatigue. (10) Qualitative work also highlights the overlapping, yet different experience of depression and fatigue after stroke. (11)

Excessive daytime sleepiness is a phenomenon related to the high likelihood of falling asleep in certain situations. It usually develops in people with sleep disorders, which are common after stroke. (12) Although daytime sleepiness and fatigue are different phenomena, sleep disorders are also associated with the development of fatigue.

Apathy is characterized by significant decrease of motivation and self-initiation. Its typical features include restricted affect and interests, loss of goal-directed behavior. Apathy is strongly associated with older age and cognitive decline. (13) One longitudinal study assessed the association between post-stroke fatigue and apathy and found no relationship. (9) However, some forms of apathy, i.e. executive apathy, characterized by lack of motivation to finish things, could be more related to post-stroke fatigue than other forms. (14)

A wide range of other conditions have fatigue as a side-effect. Therefore, clinically it is important that other potential co-morbidities or possible modifiable causes of post-stroke fatigue are not missed. Such conditions can include somatic disease (e.g. substantive unexplained weight loss, inflammatory arthritis or connective tissue disease, cardiorespiratory disease) and additional investigations may be warranted. Other causes of fatigue include endocrine disorders, anemia, kidney and liver failure, chronic inflammatory diseases, dehydration, alcohol use, and sleep apnea. The latter could be relevant in males, people with obesity, history of snoring and disrupted breathing during sleep.

**References**

1. Stroke Foundation. Clinical Guidelines for Stroke Management Melbourne, Australia. 2023.

2. Lanctôt KL, Lindsay MP, Smith EE, Sahlas DJ, Foley N, Gubitz G, et al. Canadian stroke best practice recommendations: mood, cognition and fatigue following stroke, update 2019. International Journal of Stroke. 2020;15(6):668-88.

3. Zhang S, Cheng S, Zhang Z, Wang C, Wang A, Zhu W. Related risk factors associated with post-stroke fatigue: a systematic review and meta-analysis. Neurological Sciences. 2021;42:1463-71.

4. Vitturi BK, Mitre LP, Kim AIH, Gagliardi RJ. Prevalence and predictors of fatigue and neuropsychiatric symptoms in patients with minor ischemic stroke. Journal of Stroke and Cerebrovascular Diseases. 2021;30(9):105964.

5. Huang S, Fan H, Shi Y, Hu Y, Gu Z, Chen Y. Immune biomarkers are associated with poststroke fatigue at six months in patients with ischemic stroke. Journal of Clinical Neuroscience. 2022;101:228-33.

6. Chen K, Marsh EB. Chronic post-stroke fatigue: It may no longer be about the stroke itself. Clinical neurology and neurosurgery. 2018;174:192-7.

7. Aarnes R, Stubberud J, Lerdal A. A literature review of factors associated with fatigue after stroke and a proposal for a framework for clinical utility. Neuropsychological rehabilitation. 2020;30(8):1449-76.

8. Chun H-YY, Ford A, Kutlubaev MA, Almeida OP, Mead GE. Depression, anxiety, and suicide after stroke: a narrative review of the best available evidence. Stroke. 2022;53(4):1402-10.

9. Douven E, Köhler S, Schievink SH, van Oostenbrugge RJ, Staals J, Verhey FR, et al. Temporal associations between fatigue, depression, and apathy after stroke: results of the cognition and affect after stroke, a prospective evaluation of risks study. Cerebrovascular Diseases. 2017;44(5-6):330-7.

10. Choi-Kwon S, Choi J, Kwon SU, Kang D-W, Kim JS. Fluoxetine is not effective in the treatment of poststroke fatigue: a double-blind, placebo-controlled study. Cerebrovascular Diseases. 2007;23(2-3):103-8.

11. Ablewhite J, Nouri F, Whisker A, Thomas S, Jones F, das Nair R, et al. How do stroke survivors and their caregivers manage post-stroke fatigue? A qualitative study. Clinical Rehabilitation. 2022:02692155221107738.

12. Baillieul S, Dekkers M, Brill A-K, Schmidt MH, Detante O, Pépin J-L, et al. Sleep apnoea and ischaemic stroke: current knowledge and future directions. The Lancet Neurology. 2022;21(1):78-88.

13. Tay J, Morris RG, Markus HS. Apathy after stroke: Diagnosis, mechanisms, consequences, and treatment. International Journal of Stroke. 2021;16(5):510-8.

14. Daumas L, Corbel C, Zory R, Corveleyn X, Fabre R, Robert P, et al. Associations, overlaps and dissociations between apathy and fatigue. Scientific Reports. 2022;12(1):7387.
