# Supplementary material for: A roadmap for research in post-stroke fatigue: Consensus-based core recommendations from the third Stroke Recovery and Rehabilitation Roundtable
Source: Int J Stroke. 2023 Oct 12;19(2):133–44. doi: 10.1177/17474930231189135 (PMC10811972; doi:10.1177/17474930231189135)
Supplement: sj-docx-4-wso-10.1177_17474930231189135 – Supplemental material for A roadmap for research in post-stroke fatigue: Consensus-based core recommendations from the third Stroke Recovery and Rehabilitation Roundtable [file sj-docx-4-wso-10.1177_17474930231189135.docx]

**Supplemental 4**

**Methods for intervention priority area**

Members of the working party for the intervention priority area were Gillian Mead, Avril Drummond, Coralie English, Ellyn Riley, Dale Corbett, Annapoorna Kuppuswamy, Dawn Simpson, Amreen Mahmood.

We focused on the management of post-stroke fatigue rather than its prevention because fatigue management is a leading priority for rehabilitation and life after stroke research. (1) We considered human research only because of the biopsychosocial nature of fatigue. We sought the following publication types about post-stroke fatigue management (Supplementary Table 4.1): systematic reviews of, and recently published randomised trials (RCTs) of post-stroke fatigue; clinical guidelines; ongoing RCTs; evidence from other conditions where fatigue is prevalent (Supplementary Table 1). We critically appraised and summarised this literature prior to the in-person Roundtable meeting (December 2022).

As we did not find definitive trials about post-stroke fatigue management, our discussions focused on the most promising management approaches. We focused on those approaches that aligned with potential biological mechanisms. We also discussed recommendations for future study design.

**Table 4.1 Search methods used for review of papers for promising interventions for fatigue.**

| Scope | Methods |
| --- | --- |
| Systematic reviews of, and recently published randomised controlled trials of post-stroke fatigue | Expert group (Fatigue Task Force), known recent systematic reviews were identified. We set up alerts on Medline for new trials. Fatigue Task Force members were authors on all recent published systematic reviews. |
| Clinical guidelines | Members of Fatigue Task Force are part of Guideline groups in their countries. Gillian Mead recently completed a review of 200 Clinical Guidelines worldwide on behalf of the World Stroke Organisation. |
| Ongoing trials | We searched the World Health Organisation clinical trials database (December 2022, and again in March 2023) using terms ‘fatigue AND stroke’ |
| Evidence from other conditions where fatigue is prevalent (excluding cancer related fatigue because range of different cancers, and difficulty differentiating cause of fatigue between cancer or treatments) | We searched PubMed on 8th July 2022 to retrieve systematic reviews and meta-analysis published from 2018 to 2022. The search terms used were “Covid-19”, “Myasthenia gravis”, “Rheumatoid arthritis”, “Fibromyalgia”, “Neuropathy”, “Motor Neuron Disease”, “Parkinson’s disease”, “Chronic Fatigue”, and “Multiple sclerosis”. Each of the conditions were combined with the terms “Fatigue” and “Interventions” using the Boolean ‘AND’. |

**Summary of completed trials for post-stroke fatigue:**

Several systematic reviews have been completed since 2015 exploring the effect of interventions for post-stroke fatigue. While each review had similar research questions, the studies included vary depending on whether fatigue is the primary outcome of interest and due to differences in search strategies. The identified systematic reviews were: Wu et al (2015), (2) (search dates inception to May 2014) Hinkle et al (2017) (3) (search dates Jan 2000 to March 2016) Aali (2019) (4) (search dates March 2016 to Jan 2020) and Tai et al (2022) (5) (search dates inception to 2022). We monitored for studies published after January 2020 by setting up Medline database alerts. We extracted and summarised (Table 1 in main paper) identified RCTs in which fatigue was the primary outcome or intended target of the intervention. Other trials, including several of antidepressant medication, measured fatigue as a secondary outcome. These are not included in the table 1, but of note, none of the trials of antidepressant medication reported reductions in measures of fatigue. In 2020, Xu et al (6) published a systematic review of ‘Adjuvant therapy with Astragalus membranaceus for post-stroke fatigue’. Of the 16 studies included in this systematic review, only 2 were identified in the previous systematic reviews published (indicated in the table by *). We did not extract further studies from this systematic review as the authors conclusions were that the evidence was overall poor in quality and had a high risk of bias.

**Table 4.3 Recommendations for post-stroke fatigue management in international guidelines and consensus statements**

| **Guideline** | **Recommendations for post-stroke fatigue management** | **Description of underpinning evidence** |
| --- | --- | --- |
| Australian and New Zealand Living Clinical Guidelines for Stroke Management, Stroke Foundation (7) | *Consensus-based recommendations*   - Therapy for stroke survivors with fatigue should be organised for periods of the day when they are most alert - Stroke survivors and their families/carers should be provided with information, education, and strategies to assist in managing fatigue - Potential modifying factors for fatigue should be considered, including avoiding sedating drugs and alcohol, and screening for sleep-related breathing disorders and depression - While there is insufficient evidence to guide practice, possible interventions could include cognitive behavioural therapy (focusing on fatigue and sleep with advice on regular exercise), exercise and improving sleep hygiene   Currently insufficient evidence to recommend for or against specific pharmacologic or non-pharmacologic interventions to treat fatigue in stroke survivors. Some suggested interventions include:   - Fluoxetine, enerion, fatigue education program and a mindfulness-based stress reduction program - Modafinil (further data required) - Psychologist-delivered cognitive behavioural therapy (CBT) intervention (potential value but needs further investigation) | For topics where there is either a lack of evidence or insufficient quality of evidence on which to base a recommendation, but the guideline panel believed advice should be made, *statements were developed based on consensus and expert opinion* (guided by any underlying or indirect evidence). |
| Poststroke Fatigue: Emerging Evidence and Approaches to Management, AHA Scientific Statement 2017 (3) | *Pharmacological interventions*   - Tirilazad mesylate (limited generalisability; only female participants) - Modafinil (improved fatigue in brainstem and diencephalic stroke) - OSU6162 (small RCT, needs further investigation) - Selective serotonin reuptake inhibitors (**not** effective) - Vitamin supplementation (effective but insufficient data to draw firm conclusions) - Traditional Chinese herbs such as Astragalus membranaceus (may be effective)   *Non-pharmacological interventions*   - No evidence on non-pharmacological interventions but some suggested interventions include:   - Regular exercise may help decrease PSF   - Aerobic exercises   - Activity pacing   - Patient and caregiver education on exercising, establishing good sleep patterns, avoiding sedative drugs and excessive alcohol | A critical analysis of published quantitative research and guidelines on fatigue after stroke was conducted for article published between January 2000 to March 2016. Databases  searched included PubMed, CINAHL, MEDLINE, and  PsycINFO |
| Guidelines for Adult Stroke Rehabilitation and Recovery, AHA/ASA 2016 (8) | - Not specific to fatigue but for deconditioning and fitness after stroke, individually tailored exercise prescription and physical activity is recommended (1A) - Modafinil (improved fatigue severity in brainstem and diencephalic stroke) (2B) | **Class I** Conditions for which there is evidence for and/or general agreement that the procedure or treatment is useful and effective  **Class II** Conditions for which there is conflicting evidence and/or a divergence of opinion about the usefulness /efficacy of a procedure or treatment  **Class IIa** The weight of evidence or opinion is in  favor of the procedure or treatment  **Class IIb** Usefulness  /efficacy is less well established by evidence or opinion  **Class III** Conditions for which there is evidence and/or general agreement that the procedure or treatment is not useful/effective and in some cases may be harmful  **Level A:** Multiple RCT or metanalysis  **Level B:** Single RCT or non-RCTs  **Level C**: Consensus-opinion of experts or case studies |
| Canadian Stroke Best Practice recommendations: Mood, Cognition and Fatigue Following Stroke practice guidelines, update 2020 (9) | Insufficient evidence to recommend pharmacologic or nonpharmacologic interventions   1. Management strategies for PSF can vary, and are not mutually exclusive, due to the potential multifactorial nature of PSF. In addition to education of both patient and caregivers about PSF, and treatment of any co-morbid condition that could cause/exacerbate fatigue, strategies can include the following categories:  - Strategies for energy conservation and fatigue management that take into account optimizing daily function in high priority activities (e.g. daily routines and modified tasks that anticipate energy needs and provide a balance of activity/rest) (Evidence Level C) - Engaging in planned exercise schedules with increasing physical demands appropriate to tolerance level to improve deconditioning and physical tolerance (Evidence Level C) - Education in, and establishment of, good sleep hygiene behaviors (Evidence Level B) - Communicating energy status and rest needs to family members, caregivers, employers, and social groups (Evidence Level C)  1. Stroke patients should be cared for by healthcare professionals who are knowledgeable in the symptoms of fatigue and its management (Evidence Level C) 2. There is insufficient evidence to recommend specific pharmacological treatment for PSF currently (Evidence Level B) | **Level A**: Evidence from a meta-analysis of randomized controlled trials or consistent findings from two or  more randomized controlled trials. Desirable effects clearly outweigh undesirable effects, or undesirable  effects clearly outweigh desirable effects  **Level B:** Evidence from a single randomized controlled trial or  consistent findings from two or more well-designed nonrandomized and/or noncontrolled trials, and large observational studies. Desirable effects  outweigh or are closely balanced with undesirable effects or undesirable effects outweigh or are closely balanced with desirable effects  **Level C**: Writing group consensus and/or supported by limited research evidence. Desirable effects outweigh or are closely balanced with undesirable effects or  undesirable effects outweigh or are closely balanced with desirable effects, as determined by writing group consensus. Recommendations assigned a Level C evidence may be key system drivers supporting other recommendations, and some may be expert opinion based on common, new, or emerging evidence or practice patterns |
| National Clinical Guideline for Stroke for the United Kingdom and Ireland. Royal College of Physicians 2023 (10) | 1. Healthcare professionals should anticipate post-stroke fatigue, and ask people with stroke (or their family/ carers) if they experience fatigue and how it impacts on their life 2. Healthcare professionals should use a validated measure in their assessment of post-stroke fatigue, with a clear rationale for its selection, and should also consider physical and psychological fatigue, personality style, context demands and coping styles. 3. People with stroke should be assessed and periodically reviewed for post-stroke fatigue, including for factors that might precipitate or exacerbate fatigue (e.g. depression and anxiety, sleep disorders, pain) and these factors should be addressed accordingly. Appropriate time points for review are at discharge from hospital and then at regular intervals, including at 6 months and annually thereafter. 4. People with stroke should be provided with information and education regarding fatigue being a common post-stroke problem, and with reassurance and support as early as possible, including how to prevent and manage it, and signposting to peer support and voluntary sector organisations. Information should be provided in appropriate and accessible formats. 5. People with post-stroke fatigue should be involved in decision making about strategies to prevent and manage it that are tailored to their individual needs, goals and circumstances. 6. People with post-stroke fatigue should be referred to appropriately skilled and experienced clinicians as required, and should be considered for the following approaches, whilst being aware that no single measure will be effective for everyone: 7. ‒ building acceptance and adjustment to post-stroke fatigue and recognising the need to manage it;    1. ‒ education on post-stroke fatigue for the person with stroke, and their family/ and carers;    2. ‒ using a diary to record activities and fatigue;    3. ‒ predicting situations that may precipitate or exacerbate fatigue;    4. ‒ pacing and prioritising activities;    5. ‒ relaxation and meditation;    6. ‒ rest;    7. ‒ setting small goals and gradually expanding activities;    8. ‒ changing diet and/or exercise (applied with caution and tailored to individual needs);    9. ‒ seeking peer support and/or professional advice;    10. ‒ coping methods including compensatory techniques, equipment and environmental adaptations 8. Healthcare professionals working with people affected by post-stroke fatigue should be provided with education and training on post-stroke fatigue, including its multi-factorial nature and impact, potential causes and triggers, validated assessment tools and the importance of involving people affected by post-stroke fatigue in designing strategies to prevent and manage it. | Recommendations are based on one Cochrane systematic review (Legg et al, 2019), three other systematic reviews (Mead et al, 2019; Pacheco et al, 2019; Chen et al, 2022); four randomised controlled trials (Bivard et al, 2017; Dennis et al, 2020; Hankey et al, 2020; Dong et al, 2021) and one follow-up study (Hankey et al, 2021), one survey (Ablewhite et al, 2022a), two qualitative studies (Drummond et al, 2021; Ablewhite et al, 2022b), one scientific statement (Hinkle et al, 2017), and best practice recommendations (Lanctot et al, 2020) together with the consensus of the Guideline Development Group. |
|  |  |  |

**Table 4.4 Summary of ongoing trials in post-stroke fatigue**

| **Trial registration / name of trial** | **Design** | **Country** | **Target (n=)** | **Intervention** | **Control** | **Progress** |
| --- | --- | --- | --- | --- | --- | --- |
| **Pharmacological treatments (including supplements and traditional Chinese Medicine)** | | | | | | |
| A Multisite Randomized Clinical Trial Evaluating Efficacy and Safety of BP1.3656 vs Placebo in Patients with Fatigue Following Ischemic Stroke | RCT | France, Switzerland | 54 | Drug BP1.3656 | Placebo drug | Recruiting Estimated completion June 2023 |
| Placebo-controlled randomized Clinical Trial of Perispinal Etanercept in Australian patients with chronic stroke 2020: Fatigue & muscle spasticity study | RCT | Australia | 80 | Drug Etanercept | Normal Saline for injection | Active not recruiting Estimated completion October 2022 |
| A Phase III, Multicentre, Prospective, Randomised, Placebo-controlled, Double-blind, Parallel group study to evaluate the effect of Modafinil on Debilitating Fatigue in Stroke Survivors | RCT | Australia | 300 | Drug Modafinil | Placebo | Recruiting Estimated completion December 2022 |
| **Psychoeducational interventions** | | | | | | |
| The Effect of Implementation a Therapeutic Communication Program Based on Hilgard Peplau Theory on Depression and Fatigue In Patients With Stroke | Two-group pre/ post clinical trial | Iran | 84 | 3 face-to-face communication sessions during hospitalization. One online session after discharge from the hospital | Usual ward care | Recruiting Estimated completion September 2023 |
| Too tired to recover: Evaluation of a post-stroke fatigue management guideline on the fatigue experience of stroke patients | RCT | Australia | 40 | Active fatigue management (including education and use of newly developed clinical tools) | Standard care: no education about fatigue or focus on mental fatigue in therapy. May receive some compensatory strategies for cognitive or fatigue-related symptoms | Recruiting Estimated completion March 2020 |
| Reducing fatigue after stroke: A randomised controlled trial (FASTER) | RCT | New Zealand | 200 | Six weekly standardised fatigue management group sessions (fatigue management, sleep, exercise, nutrition, and mood) | General stroke education with limited focus on post-stroke fatigue | Completed June 2022 |
| Efficacy of Cognitive-Behavioural Therapy for sleep disturbance and fatigue following stroke | RCT | Australia | 126 | Cognitive Behavioural Therapy for sleep and fatigue | Health education control condition | Active, not recruiting Estimated completion October 2023 |
| **Neuromodulation interventions** | | | | | | |
| Neuromodulation for Rehabilitation of Post-Stroke Fatigue: An rTMS Pilot Study. | RCT | United States | 60 | High frequency repetitive transcranial magnetic stimulation | Sham repetitive transcranial magnetic stimulation | Not yet recruiting Estimated completion October 2027 |
| Effects of tDCS on Post-stroke Fatigue and Inflammation | RCT | United States | 24 | Anodal transcranial direct current stimulation | Sham stimulation | Recruiting Estimated completion May 2023 |
| Effect of transcranial direct current stimulation on fatigue in stroke survivors | Single group | India | 30 | transcranial direct current stimulation | NA | Not yet recruiting Estimated completion April 2025 |
| Transcranial Direct Current Stimulation in Post-Stroke Fatigue: A Double-blind Randomised Control Trial | RCT | China | 28 | transcranial direct current stimulation | Not stated | Recruiting Estimated completion December 2025 |
| A randomized controlled double-blind study for transcranial direct current stimulation in the treatment of post stroke fatigue | RCT | China | 50 | Active transcranial direct current stimulation | Sham transcranial direct current stimulation | Not yet recruiting Estimated completion December 2024 |
| **Other interventions** | | | | | | |
| Individualized Digital Coaching Twice a Week for 12 Weeks With " Physical Activity on Prescription " (FaR) to Increase Physical Activity and Reduce Post Stroke Fatigue (PSF) | RCT | Sweden | 20 | Individualized, digital coaching: twice/week, 12 weeks with "Physical activity on prescription” to increase physical activity and reduce PSF after stroke | Routine written and verbal information about PSF and information about recommended level of physical activity | Not yet recruiting Estimated completion February 2025 |
| The Feasibility and Effects of Novel Light Therapy in Individuals with Neurological Conditions- Stroke | RCT | Australia | 58 | Green-blue light therapy, delivered using Re-Timer light therapy glasses | Sleep hygiene guidance | Not yet recruiting Estimated completion December 2023 |
| Efficacy and safety of fire dragon cupping (Huo-Long-Guan) for post-stroke fatigue | RCT | China | 45 | Routine rehab + fire dragon cupping | Conventional rehab | Recruiting Estimated completion August 2023 |
| Does a Cardiorespiratory Interval Training Program at Home Improve Post-stroke Fatigue? | RCT | Sweden | 50 | Cardiorespiratory interval training | Usual Early Supported Discharge care | Recruiting Estimated completion March 2023 |
| Personalised Physical Training Associated with Usual Management Versus Usual Management Alone on Fatigue and Recovery After Minor Stroke: Randomised Controlled Trial) | RCT | France | 160 | Physical training | No physical activity | Status unknown Estimated completion November 2019 |
| Effects of Colored Light Exposure on Sleep Disturbance, Fatigue, and Functional Outcomes Following Acute Brain Injury | RCT | United States | 52 | Exposure to daily morning colored light in the 440-485 nm wavelength range (blue light) | Exposure to daily morning colored light in the 625-740 nm wavelength range (red light) | Recruiting Estimated completion December 2022 |
| FAST: A randomised controlled study of physical activity to reduce fatigue in stroke survivors | RCT | Australia | 23 | 10-week Behavioural intervention | Education on general aspects of recovery | Completed, not yet published |
| A randomized, open-label, parallel, controlled study of Wulingshen in the treatment of poststroke fatigue | RCT | China | 40 | Oral treatment with Wuling Capsules | Oral vitamin C | Status unknown Estimated completion June 2013 |

**Table 4.5 Effect of fatigue interventions in conditions other than stroke**

Rating of effectiveness based on author conclusion. Please refer to full text for detailed results and interpretation: Orange = may be effective, green = effective. Effectiveness ratings as per published abstracts, or on additional data sourced from the full text where noted (bolded).

| **Author (date)** | **N (studies), n (participants),** | **Intervention** | **Main finding  (author conclusion)** | **Effect size metric** |
| --- | --- | --- | --- | --- |
| Post-acute Covid-19 |  |  |  |  |
| Fugazzaro et al, 2022 (25) | N = 5, n = 512 | Exercise | May be effective |  |
| Myasthenia gravis |  |  |  |  |
| Corrado et al, 2020 (26) | N = 11, n = 166 | Exercise +/- psychological support | May be effective |  |
| Rheumatoid arthritis |  |  |  |  |
| **Sezgin & Bektas, 2021 (27)** | **N = 6, n = 994** | **Education and psychological support** | **Effective** | **Hedge's g = -0.18; 95% CI = -0.3 to -0.06** |
| Fibromyalgia |  |  |  |  |
| Zheng C, Zhou T., 2022 (28) | N = 13, n = 715 | Acupuncture | No effect |  |
| Welsch et al, 2018 (29) | N = 3, n = 606 | Mirtazapine | No effect |  |
| Welsch et al, 2018 (Cochrane review) (30) | N = 18, n = 7,903 | Serotonin and noradrenaline reuptake inhibitors | No effect | SMD -0.13 (-0.18to -0.08). Low certainty evidence |
| Haugmark et al, 2019 (31) | N = 9, n = 91 | Mindfulness | No effect |  |
| Moretti et al 2018 (32) | N = 3, n = 107 | Whole body vibration | No effect |  |
| **Kundakci et al, 2021 (33)** | **N = 167, n = 11,012** | **Exercise** | **Effective** | **Mind–body and strengthening exercises improved fatigue (ES -0.77 to -1.00)** |
|  |  | **Education** | **Effective** | **Three trials (n = 251) showed improved fatigue (-0.31; 95% CI -0.55 to -0.06)** |
|  |  | Cognitive behavioural therapy | No effect |  |
|  |  | Balneotherapy (hydrotherapy)  Psychological support | No effect | (-0.23; 95% CI -0.56 to 0.09) |
|  |  | **Acupuncture** | **Effective** | **Greater improvement in fatigue compared with sham acupuncture (-0.50; 95% CI -0.90 to -0.10) and usual care (-0.41; 95% CI -0.80 to -0.01)** |
| **Bidonde et al, 2019 (Cochrane review) (34)** | **N = 21, n = 1,253** | **Exercise** | **Effective** | **Mean fatigue reduced (MD -12.93, 95% CI -17.79 to -8.07; absolute difference 13% (95% CI 8% to 18%; relative change -17.7%, (95% CI -24.4%to -11.1%). Moderate certainty evidence** |
| Estévez-López et al, 2021 (35) | N = 17, n = 1,003 | Exercise | May be effective |  |
| Andrade et al, 2018 (36) | N = 6, n = 296 | Exercise | May be effective |  |
| Motor Neurone Disease |  |  |  |  |
| Gibbons et al, 2018 (37) | N = 4, n = 86 | Modafinil  Exercise  Repetitive transcranial magnetic stimulation | Uncertain effect |  |
| Parkinson’s Disease |  |  |  |  |
| Angelopoulou et al, 2020 (38) | N = 12, n = 307 | Massage | May be effective |  |
| Wang LL et al 2022 (39) | N = 9, n = 307 | Dance therapy | No effect |  |
| Wang HT et al, 2018 (40) | N = 8, n = 1,675 | Rotigotine transdermal patch | May be effective |  |
| Chronic Fatigue Syndrome |  |  |  |  |
| Galeoto et al, 2018 (41) | N = 4, n = 377 | Physiotherapy / exercise  Cognitive behavioural therapy | May be effective |  |
| Kim DY et al, 2020 (42) | N = 56, n = 6,956 | Immunomodulator  CoQ10+NADH  Graded-exercise  Acupuncture  Abdominal tuina | May be effective |  |
| Zhang et al, 2019 (43) | N = 16, n = 1346 | Acupuncture | May be effective |  |
| Ardestani et al, 2021 (44) | N = 12, n = 564 | Mindfulness  Cognitive behavioural therapy Qigong  Yoga | May be effective |  |
| Yang J et al, 2022 (45) | N = 2, n = 68 | Ginseng | Uncertain effect |  |
| You J et al, 2021 (46) | N = 15, n = 1,030 | Moxibustion (superior to acupuncture) | May be effective |  |
| **Larun L et al, 2019 (47)** | **N = 8, n = 1,518** | **Exercise** | **Effective** | ***Compared with control:***  **Probably reduces fatigue at end of treatment (SMD −0.66, 95% CI −1.01 to −0.31; 7 studies, 840 participants; moderate certainty evidence;**  ***Compared with CBT***  **little or no difference (MD 0.20, 95% CI -1.49 to 1.89; 1 study, 298 participants; low certainty evidence)**  ***Compared with ‘pacing’***  **may slightly reduce fatigue (MD −2.00, 95% CI −3.57 to −0.43; scale 0 to 33; 1 study, 305 participants; low-certainty evidence)** |
| Maksoud et al, 2021 (48) | N = 9, n = 494 | Mitochondrial-based nutraceutical interventions | Uncertain effect |  |
| Multiple Sclerosis |  |  |  |  |
| **Harrison AM et al, 2021 (49)** | **N = 113, n = 6,909** | **Exercise** | **Effective** | **34 (12%) of trials tested interventions that aimed to reduce fatigue**  **Largest effect observed for balance exercise (SMD = −0.84, 5 studies, 124 participants).**  **All other types of exercise (general, aerobic, strengthening, mixed) all had moderate effects on fatigue** |
|  |  | **Cognitive behavioural therapy** | **Effective** | **15 studies, 594 participants. (SMD = −0.60)** |
| Moss-Morris R et al, 2021 (50) | N = 34, n = 2,434 | Exercise  Cognitive behavioural therapy | May be effective |  |
| Rooney S et al, 2019 (51) | N = 13, n = 474 | Exercise  Cognitive behavioural therapy | Uncertain effect |  |
| **Liu M et al, 2019 (52)** | **N = 14, n = 207** | **transcranial direct current stimulation** | **Effective** |  |
| Ashrafi et al, 2020 (53) | N = 8, n = 146 | transcranial direct current stimulation | May be effective |  |
| **Hsu WY et al, 2021 (54)** | **N = 17 n = 383** | **transcranial direct current stimulation** | **Effective** | **mean effect size was 0.60 (95% CI, 0.31– 0.89, p < 0.001)** |
| Salarvand S et al, 2021(55) | N = 10, n = 421 | Massage | May be effective |  |
| Andreu-Caravaca L et al, 2021 (56) | N = 43, n = 1,070 | Exercise (aerobic only) | No effect | Fatigue decreased (SMD, -0.45; P<.001). However, no significant differences when compared with the control group |
| Andreu-Caravaca et al, 2022 (57) | N = 44, n = 1,105 | Exercise (resistance only) | No effect | No significant differences between groups were observed in fatigue |
| **Cortés-Pérez et al, 2021 (58)** | **N = 12, n = 606** | **Virtual reality-based exercise therapy** | **Effective** | **RCTs (10 of 12 included in meta-analysis for fatigue)**  **SMD -0.33 (95% CI -0.6 to -0.0.6)** |
| Phyo AZZ et al, 2018 (59) | N = 20, n = 1249 | Cognitive behavioural therapy Mindfulness | May be effective |  |
| Shohani M et al, 2020 (60) | N = 10, n = 693 | Yoga  Exercise | May be effective |  |
| Mohebbirad et al, 2022 (61) | N = 9, n = 357 | Vestibular retraining Sensory therapy | May be effective |  |

NR: not reported; ES: Effect Size; SMD: Standard Mean Difference.

**References**

1. Hill G, Regan S, Francis R, Mead G, Thomas S, Salman RA-S, et al. Research priorities to improve stroke outcomes. The Lancet Neurology. 2022;21(4):312-3.

2. Wu S, Kutlubaev MA, Chun H-YY, Cowey E, Pollock A, Macleod MR, et al. Interventions for post-stroke fatigue. The Cochrane database of systematic reviews. 2015(7):CD007030.

3. Hinkle JL, Becker KJ, Kim JS, Choi-Kwon S, Saban KL, McNair N, et al. Poststroke Fatigue: Emerging Evidence and Approaches to Management: A Scientific Statement for Healthcare Professionals From the American Heart Association. Stroke. 2017;48(7):e159-e70.

4. Aali G, Drummond A, das Nair R, Shokraneh F. Post-stroke fatigue: a scoping review. F1000Research. 2020;9.

5. Tai D, Falck RS, Davis JC, Vint Z, Liu‐Ambrose T. Can exercise training promote better sleep and reduced fatigue in people with chronic stroke? A systematic review. Journal of Sleep Research. 2022;31(6):e13675.

6. Xu L, Xu X-Y, Hou X-Q, Wang F-G, Gao S, Zhang H-T. Adjuvant therapy with Astragalus membranaceus for post-stroke fatigue: a systematic review. Metabolic Brain Disease. 2020;35(1):83-93.

7. Stroke Foundation. Clinical Guidelines for Stroke Management Melbourne, Australia. 2023.

8. Winstein CJ, Stein J, Arena R, Bates B, Cherney LR, Cramer SC, et al. Guidelines for Adult Stroke Rehabilitation and Recovery: A Guideline for Healthcare Professionals From the American Heart Association/American Stroke Association. Stroke. 2016;47(6):e98-e169.

9. Lanctot KL, Lindsay MP, Smith EE, Sahlas DJ, Foley N, Gubitz G, et al. Canadian Stroke Best Practice Recommendations: Mood, Cognition and Fatigue following Stroke, 6th edition update 2019. International journal of stroke : official journal of the International Stroke Society. 2020;15(6):668-88.

10. Physicians RCo. National Clinical Guideline for Stroke for the United Kingdom and Ireland London2023 [Clinical Guideline]. Available from: <https://www.strokeguideline.org/>

25. Fugazzaro S, Contri A, Esseroukh O, Kaleci S, Croci S, Massari M, Facciolongo NC, Besutti G, Iori M, Salvarani C, Costi S. Rehabilitation interventions for post-acute COVID-19 syndrome: a systematic review. International journal of environmental research and public health. 2022 Apr 24;19(9):5185.

26. Corrado B, Giardulli B, Costa M. Evidence-based practice in rehabilitation of myasthenia gravis. A systematic review of the literature. Journal of Functional Morphology and Kinesiology. 2020 Sep 27;5(4):71.

27. Sezgin MG, Bektas H. The effect of nurse‐led care on fatigue in patients with rheumatoid arthritis: A systematic review and meta‐analysis of randomised controlled studies. Journal of Clinical Nursing. 2022 Apr;31(7-8):832-42.

28. Zheng C, Zhou T. Effect of acupuncture on pain, fatigue, sleep, physical function, stiffness, well-being, and safety in fibromyalgia: a systematic review and meta-analysis. Journal of Pain Research. 2022 Feb 3:315-29.

29. Welsch P, Bernardy K, Derry S, Moore RA, Häuser W. Mirtazapine for fibromyalgia in adults. Cochrane Database Syst Rev. 2018 Aug 6;8(8):CD012708. doi: 10.1002/14651858.CD012708

30. Welsch P, Üçeyler N, Klose P, Walitt B, Häuser W. Serotonin and noradrenaline reuptake inhibitors (SNRIs) for fibromyalgia. Cochrane Database Syst Rev. 2018 Feb 28;2(2):CD010292. doi: 10.1002/14651858.CD010292.

31. Haugmark T, Hagen KB, Smedslund G, Zangi HA. Mindfulness-and acceptance-based interventions for patients with fibromyalgia–A systematic review and meta-analyses. PloS one. 2019 Sep 3;14(9):e0221897.

32. Moretti E, Tenório A, Holanda L, Campos A, Lemos A. Efficacy of the whole-body vibration for pain, fatigue and quality of life in women with fibromyalgia: a systematic review. Disabil Rehabil. 2018 May;40(9):988-996. doi: 10.1080/09638288.2017.1282989.

33. Kundakci B, Kaur J, Goh SL, Hall M, Doherty M, Zhang W, Abhishek A. Efficacy of nonpharmacological interventions for individual features of fibromyalgia: a systematic review and meta-analysis of randomised controlled trials. Pain. 2022 Aug 1;163(8):1432-1445. doi: 10.1097/j.pain.0000000000002500.

34. Bidonde J, Busch AJ, Schachter CL, Webber SC, Musselman KE, Overend TJ, Góes SM, Dal Bello‐Haas V, Boden C, Cochrane Musculoskeletal Group. Mixed exercise training for adults with fibromyalgia. Cochrane Database of Systematic Reviews. 1996 Sep 1;2019(5).

35. Estévez-López F, Maestre-Cascales C, Russell D, Alvarez-Gallardo IC, Rodriguez-Ayllon M, Hughes CM, Davison GW, Sanudo B, McVeigh JG. Effectiveness of exercise on fatigue and sleep quality in fibromyalgia: a systematic review and meta-analysis of randomized trials. Archives of Physical Medicine and Rehabilitation. 2021 Apr 1;102(4):752-61.

36. Andrade A, Steffens RD, Sieczkowska SM, Tartaruga LA, Vilarino GT. A systematic review of the effects of strength training in patients with fibromyalgia: clinical outcomes and design considerations. Advances in Rheumatology. 2019 Jul 29;58.

37. Gibbons C, Pagnini F, Friede T, Young CA. Treatment of fatigue in amyotrophic lateral sclerosis/motor neuron disease. Cochrane Database of Systematic Reviews 2018, Issue 1. Art. No.: CD011005. DOI: 10.1002/14651858.CD011005

38. Angelopoulou E, Anagnostouli M, Chrousos GP, Bougea A. Massage therapy as a complementary treatment for Parkinson’s disease: A Systematic Literature Review. Complementary therapies in medicine. 2020 Mar 1;49:102340.

39. Wang LL, Sun CJ, Wang Y, Zhan TT, Yuan J, Niu CY, Yang J, Huang S, Cheng L. Effects of dance therapy on non-motor symptoms in patients with Parkinson’s disease: a systematic review and meta-analysis. Aging Clinical and Experimental Research. 2022 Jun;34(6):1201-8.

40. Wang HT, Wang L, He Y, Yu G. Rotigotine transdermal patch for the treatment of neuropsychiatric symptoms in Parkinson's disease: A meta-analysis of randomized placebo-controlled trials. J Neurol Sci. 2018 Oct 15;393:31-38. doi: 10.1016/j.jns.2018.08.003. Epub 2018 Aug 3. PMID: 30099246.

41. Galeoto G, Sansoni J, Valenti D, Mollica R, Valente D, Parente M, Servadio A. The effect of physiotherapy on fatigue and physical functioning in chronic fatigue syndrome patients: A systematic review. Clin Ter. 2018 Jul-Aug;169(4):e184-e188. doi: 10.7417/T.2018.2076.

42. Kim DY, Lee JS, Park SY, Kim SJ, Son CG. Systematic review of randomized controlled trials for chronic fatigue syndrome/myalgic encephalomyelitis (CFS/ME). J Transl Med. 2020 Jan 6;18(1):7. doi: 10.1186/s12967-019-02196-9. Erratum in: J Transl Med. 2020 Dec 23;18(1):492.

43. Zhang Q, Gong J, Dong H, Xu S, Wang W, Huang G. Acupuncture for chronic fatigue syndrome: a systematic review and meta-analysis. Acupunct Med. 2019 Aug;37(4):211-222. doi: 10.1136/acupmed-2017-011582. Epub 2019 Jun 17. PMID: 31204859.

44. Khanpour Ardestani S, Karkhaneh M, Stein E, Punja S, Junqueira DR, Kuzmyn T, Pearson M, Smith L, Olson K, Vohra S. Systematic Review of Mind-Body Interventions to Treat Myalgic Encephalomyelitis/Chronic Fatigue Syndrome. Medicina (Kaunas). 2021 Jun 24;57(7):652. doi: 10.3390/medicina57070652. PMID: 34202826; PMCID: PMC8305555.

45. Yang J, Shin KM, Abu Dabrh AM, Bierle DM, Zhou X, Bauer BA, Mohabbat AB. Ginseng for the Treatment of Chronic Fatigue Syndrome: A Systematic Review of Clinical Studies. Glob Adv Health Med. 2022 Feb 14;11:2164957X221079790. doi: 10.1177/2164957X221079790.

46. You J, Ye J, Li H, Ye W, Hong E. Moxibustion for Chronic Fatigue Syndrome: A Systematic Review and Meta-Analysis. Evid Based Complement Alternat Med. 2021 Nov 11;2021:6418217. doi: 10.1155/2021/6418217. PMID: 34804182; PMCID: PMC8601810.

47. Larun L, Brurberg KG, Odgaard-Jensen J, Price JR. Exercise therapy for chronic fatigue syndrome. Cochrane Database Syst Rev. 2019 Oct 2;10(10):CD003200. doi: 10.1002/14651858.CD003200.pub8.

48. Maksoud R, Balinas C, Holden S, Cabanas H, Staines D, Marshall-Gradisnik S. A systematic review of nutraceutical interventions for mitochondrial dysfunctions in myalgic encephalomyelitis/chronic fatigue syndrome. J Transl Med. 2021 Feb 17;19(1):81. doi: 10.1186/s12967-021-02742-4.

49. Harrison AM, Safari R, Mercer T, Picariello F, van der Linden ML, White C, Moss-Morris R, Norton S. Which exercise and behavioural interventions show most promise for treating fatigue in multiple sclerosis? A network meta-analysis. Mult Scler. 2021 Oct;27(11):1657-1678. doi: 10.1177/1352458521996002. Epub 2021 Apr 20. Erratum in: Mult Scler. 2021 Jul 23;:13524585211034450.

50. Moss-Morris R, Harrison AM, Safari R, Norton S, van der Linden ML, Picariello F, Thomas S, White C, Mercer T. Which behavioural and exercise interventions targeting fatigue show the most promise in multiple sclerosis? A systematic review with narrative synthesis and meta-analysis. Behaviour research and therapy. 2021 Feb 1;137:103464.

51. Rooney S, Moffat F, Wood L, Paul L. Effectiveness of fatigue management interventions in reducing severity and impact of fatigue in people with progressive multiple sclerosis: A systematic review. International Journal of MS Care. 2019;21(1):35-46.

52. Liu M, Fan S, Xu Y, Cui L. Non-invasive brain stimulation for fatigue in multiple sclerosis patients: A systematic review and meta-analysis. Multiple sclerosis and related disorders. 2019 Nov 1;36:101375.

53. Ashrafi A, Mohseni-Bandpei MA, Seydi M. The effect of tDCS on the fatigue in patients with multiple sclerosis: A systematic review of randomized controlled clinical trials. Journal of Clinical Neuroscience. 2020 Aug 1;78:277-83.

54. Hsu WY, Cheng CH, Zanto TP, Gazzaley A, Bove RM. Effects of transcranial direct current stimulation on cognition, mood, pain, and fatigue in multiple sclerosis: a systematic review and meta-analysis. Frontiers in Neurology. 2021 Mar 8;12:626113.

55. Salarvand S, Heidari ME, Farahi K, Teymuri E, Almasian M, Bitaraf S. Effectiveness of massage therapy on fatigue and pain in patients with multiple sclerosis: A systematic review and meta-analysis. Multiple Sclerosis Journal–Experimental, Translational and Clinical. 2021 Jun;7(2):20552173211022779.

56. Andreu-Caravaca L, Ramos-Campo DJ, Chung LH, Rubio-Arias JÁ. Dosage and Effectiveness of Aerobic Training on Cardiorespiratory Fitness, Functional Capacity, Balance, and Fatigue in People With Multiple Sclerosis: A Systematic Review and Meta-Analysis. Arch Phys Med Rehabil. 2021 Sep;102(9):1826-1839. doi: 10.1016/j.apmr.2021.01.078.

57. Andreu-Caravaca L, Ramos-Campo DJ, Chung LH, Martínez-Rodríguez A, Rubio-Arias JÁ. Effects and optimal dosage of resistance training on strength, functional capacity, balance, general health perception, and fatigue in people with multiple sclerosis: a systematic review and meta-analysis. Disabil Rehabil. 2022 May 17:1-13. doi: 10.1080/09638288.2022.2069295.

58. Cortés-Pérez I, Sánchez-Alcalá M, Nieto-Escámez FA, Castellote-Caballero Y, Obrero-Gaitán E, Osuna-Pérez MC. Virtual reality-based therapy improves fatigue, impact, and quality of life in patients with multiple sclerosis. A systematic review with a meta-analysis. Sensors. 2021 Jan;21(21):7389.

59. Phyo AZ, Demaneuf T, De Livera AM, Jelinek GA, Brown CR, Marck CH, Neate SL, Taylor KL, Mills T, O’Kearney E, Karahalios A. The efficacy of psychological interventions for managing fatigue in people with multiple sclerosis: a systematic review and meta-analysis. Frontiers in neurology. 2018 Apr 4;9:149.

60. Shohani M, Kazemi F, Rahmati S, Azami M. The effect of yoga on the quality of life and fatigue in patients with multiple sclerosis: A systematic review and meta-analysis of randomized clinical trials. Complementary therapies in clinical practice. 2020 May 1;39:101087.

61. Mohebbirad M, Motaharinezhad F, Shahsavary M, Joveini G. Effects of Sensory Interventions on Fatigue in People With Multiple Sclerosis: A Systematic Review. International Journal of MS Care. 2022 Jan 1;24(1):29-34.
