# Supplementary material for: A roadmap for research in post-stroke fatigue: Consensus-based core recommendations from the third Stroke Recovery and Rehabilitation Roundtable
Source: Int J Stroke. 2023 Oct 12;19(2):133–44. doi: 10.1177/17474930231189135 (PMC10811972; doi:10.1177/17474930231189135)
Supplement: sj-docx-5-wso-10.1177_17474930231189135 – Supplemental material for A roadmap for research in post-stroke fatigue: Consensus-based core recommendations from the third Stroke Recovery and Rehabilitation Roundtable [file sj-docx-5-wso-10.1177_17474930231189135.docx]

**Supplemental 5**

**Methods for mechanisms priority topic area**

Additional advisory group members were recruited to form a ‘mechanisms working party’, chaired by Dale Corbett and Annapoorna Kuppuswamy. The full working party (including advisory members) for the mechanism’s topic were Dale Corbett, Anna Kuppuswamy, Mansur Kutlubaev, Lorimer Moseley, Sandra Billinger, Quentin Pittman, Connie Wong, Brad Sutherland, Kirsten Coupland, Brad Aouizerat, Andrew Bivard.

**Declaration of funding:** Lorimer Moseley is supported by a Leadership Investigator Grant from the National Health & Medical Research Council of Australia (ID1178444).

Over several months the group identified 12 topics as candidates for further study into the mechanisms of post-stroke fatigue. The taskforce and advisory group members (n=12) rated the potential importance of pursuing each of these topics on a 5-point scale with respect to increasing our understanding of post-stroke fatigue mechanisms. The topics with moderate-high rankings (score of 3-5) comprise the list of 7 topics shown below. Other topics such as use of animal models, role of classical conditioning/learning and others were not pursued due to paucity of literature.

1. Comprehensive definitions of mechanisms (i.e. cellular, system and behavioural levels)

2. Post-stroke fatigue subtypes and lesion location

3. Fatigue in other diseases

4. Biomarkers of post-stroke fatigue

5. Post-stroke Fatigue and Inflammation/Immune Dysregulation

6. Dopamine and post-stroke fatigue

7. Exercise and post-stroke fatigue

Via a process of targeted literature reviews, the 7 topics were investigated in depth for their potential role and/or relevance to post-stroke fatigue and for guiding future fatigue research. A small team (2-4 people depending on topic) reviewed literature, discussed findings, and then wrote a summary draft of their work. These were circulated and with input from all working party members led by the co-chairs (DC and AK), led to several revised versions of each document. These were then compiled into the single document for review and discussion at the face-to-face meeting.

**Bibliography**:

1. Chaudhuri A, Behan PO. Fatigue in neurological disorders. Lancet. 2004;363(9413):978-988. doi:10.1016/S0140-6736(04)15794-2

2. Booij HA, Gaykema WDC, Kuijpers K a. J, Pouwels MJM, den Hertog HM. Pituitary dysfunction and association with fatigue in stroke and other acute brain injury. Endocr Connect. 2018;7(6):R223-R237. doi:10.1530/EC-18-0147

3. Manjaly ZM, Harrison NA, Critchley HD, et al. Pathophysiological and cognitive mechanisms of fatigue in multiple sclerosis. J Neurol Neurosurg Psychiatry. Published online January 25, 2019. doi:10.1136/jnnp-2018-320050

4. Bhandari K, Kapoor D. Fatigue in Cirrhosis. J Clin Exp Hepatol. 2022;12(2):617-624. doi:10.1016/j.jceh.2021.08.028

5. Fatigue in inflammatory rheumatic diseases: current knowledge and areas for future research - PubMed. Accessed February 20, 2023. https://pubmed.ncbi.nlm.nih.gov/34599320/

6. Eriksson G, Larsson I, Guidetti S, Johansson U. Handling fatigue in everyday activities at five years after stroke: A long and demanding process. Scand J Occup Ther. Published online June 25, 2022:1-11. doi:10.1080/11038128.2022.2089230

7. Ablewhite J, Nouri F, Whisker A, et al. How do stroke survivors and their caregivers manage post-stroke fatigue? A qualitative study. Clin Rehabil. Published online June 30, 2022:2692155221107738. doi:10.1177/02692155221107738

8. Delva M, Lytvynenko N, Delva I. FACTORS ASSOCIATED WITH THE TIME-BASED PHENOMENOLOGY OF POST-STROKE FATIGUE OVER THE FIRST YEAR AFTER STROKE OCCURRENCE. Georgian Med News. 2018;(279):92-97.

9. Hubacher M, Calabrese P, Bassetti C, Carota A, Stöcklin M, Penner IK. Assessment of post-stroke fatigue: the fatigue scale for motor and cognitive functions. Eur Neurol. 2012;67(6):377-384. doi:10.1159/000336736

10. Schow T, Teasdale TW, Quas KJ, Rasmussen MA. Problems with balance and binocular visual dysfunction are associated with post-stroke fatigue. Top Stroke Rehabil. 2017;24(1):41-49. doi:10.1080/10749357.2016.1188475

11. Johansson B, Rönnbäck L. Mental fatigue and cognitive impairment after an almost neurological recovered stroke. ISRN Psychiatry. 2012;2012:686425. doi:10.5402/2012/686425

12. Doncker WD, Ondobaka S, Kuppuswamy A. Effect of transcranial direct current stimulation on post-stroke fatigue. medRxiv. Published online November 20, 2020:2020.11.18.20227272. doi:10.1101/2020.11.18.20227272

13. Zedlitz AMEE, Rietveld TCM, Geurts AC, Fasotti L. Cognitive and graded activity training can alleviate persistent fatigue after stroke: a randomized, controlled trial. Stroke. 2012;43(4):1046-1051. doi:10.1161/STROKEAHA.111.632117

14. Bivard A, Lillicrap T, Krishnamurthy V, et al. MIDAS (Modafinil in Debilitating Fatigue After Stroke): A Randomized, Double-Blind, Placebo-Controlled, Cross-Over Trial. Stroke. 2017;48(5):1293-1298. doi:10.1161/STROKEAHA.116.016293

15. Lewis SJ, Barugh AJ, Greig CA, et al. Is fatigue after stroke associated with physical deconditioning? A cross-sectional study in ambulatory stroke survivors. Arch Phys Med Rehabil. 2011;92(2):295-298. doi:10.1016/j.apmr.2010.10.030

16. Ponchel A, Bombois S, Bordet R, Hénon H. Factors Associated with Poststroke Fatigue: A Systematic Review. Stroke Research and Treatment. doi:https://doi.org/10.1155/2015/347920

17. Appelros P. Prevalence and predictors of pain and fatigue after stroke: a population-based study. Int J Rehabil Res. 2006;29(4):329-333. doi:10.1097/MRR.0b013e328010c7b8

18. Naess H, Lunde L, Brogger J, Waje-Andreassen U. Fatigue among stroke patients on long-term follow-up. The Bergen Stroke Study. J Neurol Sci. 2012;312(1-2):138-141. doi:10.1016/j.jns.2011.08.002

19. Zhang S, Cheng S, Zhang Z, Wang C, Wang A, Zhu W. Related risk factors associated with post-stroke fatigue: a systematic review and meta-analysis. Neurol Sci. Published online August 19, 2020. doi:10.1007/s10072-020-04633-w

20. Wang J, Gu M, Xiao L, et al. Association of Lesion Location and Fatigue Symptoms After Ischemic Stroke: A VLSM Study. Front Aging Neurosci. 2022;14:902604. doi:10.3389/fnagi.2022.902604

21. Bonkhoff AK, Xu T, Nelson A, et al. Reclassifying stroke lesion anatomy. Cortex. 2021;145:1-12. doi:10.1016/j.cortex.2021.09.007

22. Analysis of Factors Affecting Post-Stroke Fatigue: An Observational, Cross-Sectional, Retrospective Chart Review Study - PubMed. Accessed February 20, 2023. https://pubmed.ncbi.nlm.nih.gov/34828631/

23. Huang S, Fan H, Shi Y, Hu Y, Gu Z, Chen Y. Immune biomarkers are associated with poststroke fatigue at six months in patients with ischemic stroke. J Clin Neurosci. 2022;101:228-233. doi:10.1016/j.jocn.2022.05.020

24. Depressed TSH level as a predictor of poststroke fatigue in patients with acute ischemic stroke - PubMed. Accessed February 20, 2023. https://pubmed.ncbi.nlm.nih.gov/30366976/

25. Ormstad H, Aass HCD, Amthor KF, Lund-Sørensen N, Sandvik L. Serum levels of cytokines, glucose, and hemoglobin as possible predictors of poststroke depression, and association with poststroke fatigue. Int J Neurosci. 2012;122(11):682-690. doi:10.3109/00207454.2012.709892

26. Ormstad H, Aass HCD, Amthor KF, Lund-Sørensen N, Sandvik L. Serum cytokine and glucose levels as predictors of poststroke fatigue in acute ischemic stroke patients. J Neurol. 2011;258(4):670-676. doi:10.1007/s00415-011-5962-8

27. Klinedinst NJ, Schuh R, Kittner SJ, et al. Post-stroke fatigue as an indicator of underlying bioenergetics alterations. J Bioenerg Biomembr. 2019;51(2):165-174. doi:10.1007/s10863-018-9782-8

28. Kutlubaev MA, Duncan FH, Mead GE. Biological correlates of post-stroke fatigue: a systematic review. Acta Neurol Scand. 2012;125(4):219-227. doi:10.1111/j.1600-0404.2011.01618.x

29. Liu X, Wang B, Wang X, Tian M, Wang X, Zhang Y. Elevated plasma high-sensitivity C-reactive protein at admission predicts the occurrence of post-stroke fatigue at 6 months after ischaemic stroke. Eur J Neurol. 2020;27(10):2022-2030. doi:10.1111/ene.14430

30. McKechnie F, Lewis S, Mead G. A pilot observational study of the association between fatigue after stroke and C-reactive protein. J R Coll Physicians Edinb. 2010;40(1):9-12. doi:10.4997/JRCPE.2010.103

31. Wen H, Weymann KB, Wood L, Wang QM. Inflammatory Signaling in Post-Stroke Fatigue and Depression. Eur Neurol. 2018;80(3-4):138-148. doi:10.1159/000494988

32. Grossberg AJ, Zhu X, Leinninger GM, et al. Inflammation-induced lethargy is mediated by suppression of orexin neuron activity. J Neurosci. 2011;31(31):11376-11386. doi:10.1523/JNEUROSCI.2311-11.2011

33. Gyawali P, Hinwood M, Chow WZ, et al. Exploring the relationship between fatigue and circulating levels of the pro-inflammatory biomarkers interleukin-6 and C-reactive protein in the chronic stage of stroke recovery: A cross-sectional study. Brain Behav Immun Health. 2020;9:100157. doi:10.1016/j.bbih.2020.100157

34. Becker K, Kohen R, Lee R, et al. Poststroke fatigue: hints to a biological mechanism. J Stroke Cerebrovasc Dis. 2015;24(3):618-621. doi:10.1016/j.jstrokecerebrovasdis.2014.10.008

35. Kuppuswamy A. The Neurobiology of Pathological Fatigue: New Models, New Questions. Neuroscientist. Published online January 15, 2021:1073858420985447. doi:10.1177/1073858420985447

36. Kuppuswamy A. Role of selective attention in fatigue in neurological disorders. Eur J Neurol. Published online February 11, 2023. doi:10.1111/ene.15739

37. Kuppuswamy A. The fatigue conundrum. Brain. 2017;140(8):2240-2245. doi:10.1093/brain/awx153

38. Kuppuswamy A, Clark EV, Turner IF, Rothwell JC, Ward NS. Post-stroke fatigue: a deficit in corticomotor excitability? Brain. 2015;138(Pt 1):136-148. doi:10.1093/brain/awu306

39. De Doncker W, Brown KE, Kuppuswamy A. Influence of post-stroke fatigue on reaction times and corticospinal excitability during movement preparation. Clinical Neurophysiology. 2021;132(1):191-199. doi:10.1016/j.clinph.2020.11.012

40. Ondobaka S, De Doncker W, Ward N, Kuppuswamy A. Neural effective connectivity explains subjective fatigue in stroke. Brain. Published online November 17, 2021:awab287. doi:10.1093/brain/awab287

41. Wu CH, Doncker WD, Croce P, Bertoli M, Tecchio F, Kuppuswamy A. Electroencephalography-derived functional connectivity in sensorimotor networks in Stroke and Multiple Sclerosis Fatigue. Published online March 18, 2022:2022.03.16.484592. doi:10.1101/2022.03.16.484592

42. Doncker WD, Kuppuswamy A. The Cocktail Party Effect in Post-Stroke Fatigue: an EEG study. Published online March 19, 2022:2022.03.17.484808. doi:10.1101/2022.03.17.484808

43. Kuppuswamy A, Harris AM, Doncker WD, Alexander A, Lavie N. Diminished distractor filtering with increased perceptual load and sustained effort explains attention deficit in post-stroke fatigue. Published online March 19, 2022:2022.03.17.484709. doi:10.1101/2022.03.17.484709

44. Brioschi A, Gramigna S, Werth E, et al. Effect of modafinil on subjective fatigue in multiple sclerosis and stroke patients. Eur Neurol. 2009;62(4):243-249. doi:10.1159/000232927

45. Billinger SA, Arena R, Bernhardt J, et al. Physical activity and exercise recommendations for stroke survivors: a statement for healthcare professionals from the American Heart Association/American Stroke Association. Stroke. 2014;45(8):2532-2553. doi:10.1161/STR.0000000000000022

46. Ploughman M, Austin MW, Glynn L, Corbett D. The effects of poststroke aerobic exercise on neuroplasticity: a systematic review of animal and clinical studies. Transl Stroke Res. 2015;6(1):13-28. doi:10.1007/s12975-014-0357-7

47. Tai D, Falck RS, Davis JC, Vint Z, Liu-Ambrose T. Can exercise training promote better sleep and reduced fatigue in people with chronic stroke? A systematic review. J Sleep Res. 2022;31(6):e13675. doi:10.1111/jsr.13675

48. Jeffers MS, Karthikeyan S, Gomez-Smith M, et al. Does Stroke Rehabilitation Really Matter? Part B: An Algorithm for Prescribing an Effective Intensity of Rehabilitation. Neurorehabil Neural Repair. 2018;32(1):73-83. doi:10.1177/1545968317753074

49. Hayward KS, Churilov L, Dalton EJ, et al. Advancing Stroke Recovery Through Improved Articulation of Nonpharmacological Intervention Dose. Stroke. 2021;52(2):761-769. doi:10.1161/STROKEAHA.120.032496

50. Gubert C, Hannan AJ. Exercise mimetics: harnessing the therapeutic effects of physical activity. Nat Rev Drug Discov. 2021;20(11):862-879. doi:10.1038/s41573-021-00217-1

Missing/additional references

51. Lacourt, TE, et al. The High Costs of Low-Grade Inflammation: Persistent Fatigue as a Consequence of Reduced Cellular-Energy Availability and Non-adaptive Energy Expenditure Front Behav Neurosci. 2018;12: doi: 10.3389/fnbeh.2018.00078

52. Wang H, Zhang M, Li J, Liang J, Yang M, et al. Gut microbiota is causally associated with poststroke cognitive impairment through lipopolysaccharide and butyrate. J Neuroinflamm 2022; 19: 76. doi.org/10.1186/s12974-022-02435-9

53. Dobryakova E, Genova HM, DeLuca J, Wykie GR. The dopamine imbalance hypothesis of fatigue in multiple sclerosis and other neurological disorders. Front Neurol 2015; doi: 10.3389/fneur.2015.00052

54. Kip E, Parr-Brownlie LC. Healthy lifestyles and wellbeing reduce neuroinflammation and prevent neurodegenerative and psychiatric disorders. Front Neurosci 2023; DOI 10.3389/fnins.2023.1092537

55. Sommer CJ, Schabitz W-R. Fostering post-stroke recovery Towards Combination treatments. Stroke 2017; 48: 112-1119. DOI: 10.1161/STROKEAHA.116.013324

56. Ermine CM, Nithianantharajah J, O’Brien K, Kauhausen JA, Frausin S. Hemispheric cortical atrophy and chronic microglial activation following mild focal ischemic stroke in adult male rats. J Neurosci Res 2021; DOI: 10.1002/jnr.24939
