# Supplementary material for: A roadmap for research in post-stroke fatigue: Consensus-based core recommendations from the third Stroke Recovery and Rehabilitation Roundtable
Source: Int J Stroke. 2023 Oct 12;19(2):133–44. doi: 10.1177/17474930231189135 (PMC10811972; doi:10.1177/17474930231189135)
Supplement: sj-pptx-6-wso-10.1177_17474930231189135 – Supplemental material for A roadmap for research in post-stroke fatigue: Consensus-based core recommendations from the third Stroke Recovery and Rehabilitation Roundtable [file sj-pptx-6-wso-10.1177_17474930231189135.pptx]

## Slide 1
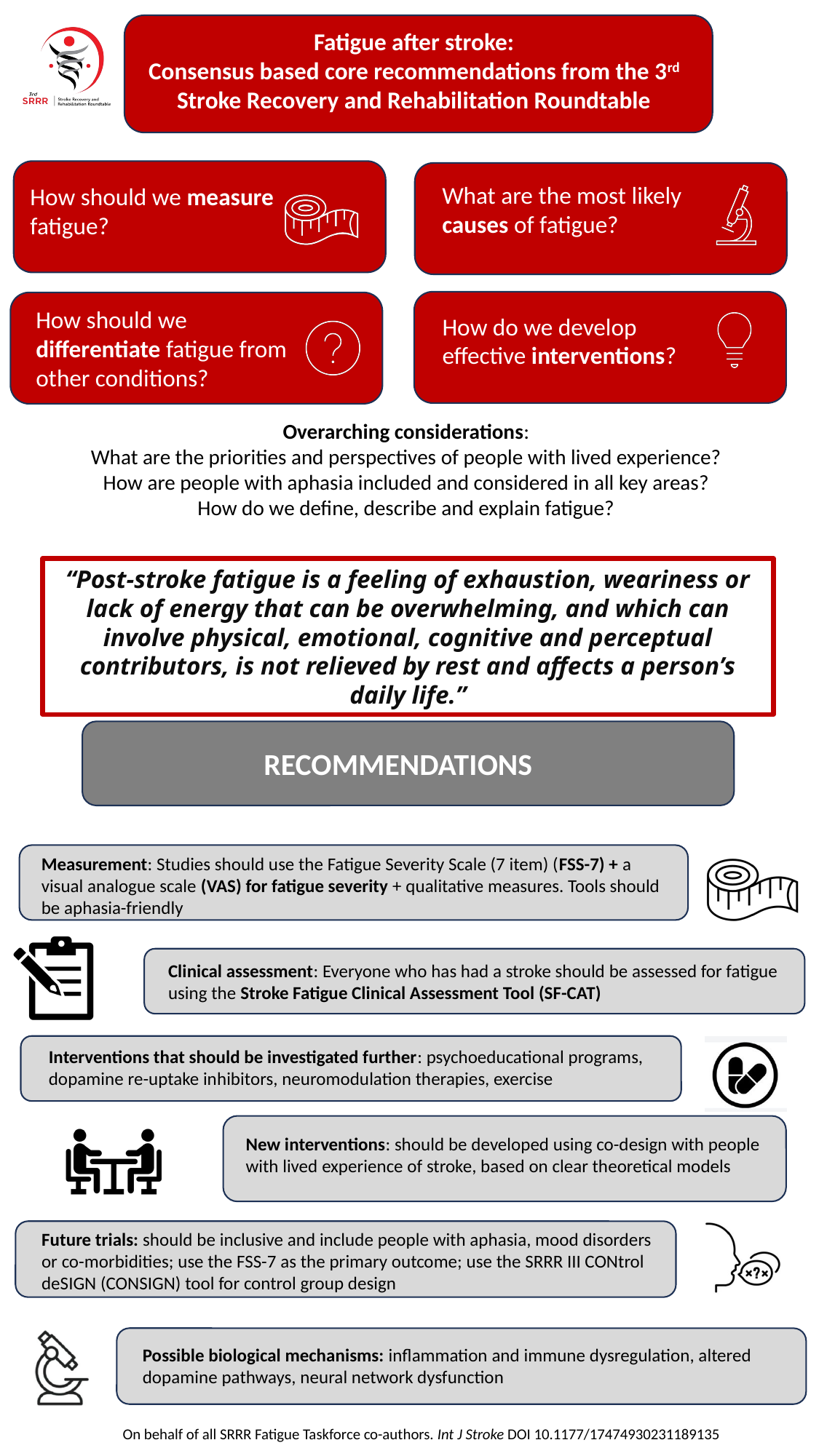

Fatigue after stroke:
Consensus based core recommendations from the 3rd Stroke Recovery and Rehabilitation Roundtable
What are the most likely causes of fatigue?
How should we measure fatigue?
How should we differentiate fatigue from other conditions?
How do we develop effective interventions?
Overarching considerations:
What are the priorities and perspectives of people with lived experience?
How are people with aphasia included and considered in all key areas?
How do we define, describe and explain fatigue?
“Post-stroke fatigue is a feeling of exhaustion, weariness or lack of energy that can be overwhelming, and which can involve physical, emotional, cognitive and perceptual contributors, is not relieved by rest and affects a person’s daily life.”
RECOMMENDATIONS
Measurement: Studies should use the Fatigue Severity Scale (7 item) (FSS-7) + a visual analogue scale (VAS) for fatigue severity + qualitative measures. Tools should be aphasia-friendly
Clinical assessment: Everyone who has had a stroke should be assessed for fatigue using the Stroke Fatigue Clinical Assessment Tool (SF-CAT)
Interventions that should be investigated further: psychoeducational programs, dopamine re-uptake inhibitors, neuromodulation therapies, exercise
New interventions: should be developed using co-design with people with lived experience of stroke, based on clear theoretical models
Future trials: should be inclusive and include people with aphasia, mood disorders or co-morbidities; use the FSS-7 as the primary outcome; use the SRRR III CONtrol deSIGN (CONSIGN) tool for control group design
Possible biological mechanisms: inflammation and immune dysregulation, altered dopamine pathways, neural network dysfunction
On behalf of all SRRR Fatigue Taskforce co-authors. Int J Stroke DOI 10.1177/17474930231189135
